# HINT1 suppression protects against age-related cardiac dysfunction by enhancing mitochondrial biogenesis

**DOI:** 10.1016/j.molmet.2025.102107

**Published:** 2025-02-03

**Authors:** Michio Sato, Tsuyoshi Kadomatsu, Jun Morinaga, Yuya Kinoshita, Daisuke Torigoe, Haruki Horiguchi, Sumio Ohtsuki, Shuji Yamamura, Ryoko Kusaba, Takanori Yamaguchi, Goro Yoshioka, Kimi Araki, Tomohiko Wakayama, Keishi Miyata, Koichi Node, Yuichi Oike

**Affiliations:** 1 Department of Molecular Genetics, Kumamoto University, Kumamoto, Japan; 2 Center for Metabolic Regulation of Healthy Aging (CMHA), Graduate School of Medical Sciences, Kumamoto University, Kumamoto, Japan; 3 Division of Kumamoto Mouse Clinic (KMC), Institute of Resource Developmental and Analysis (IRDA), Kumamoto University, Kumamoto, Japan; 4 Department of Cardiovascular Medicine, School of Medicine, Saga University, Saga, Japan; 5 Division of Experimental Genetics, IRDA, Kumamoto University, Kumamoto, Japan; 6 Department of Aging and Geriatric Medicine, Graduate School of Medical Sciences, Kumamoto University, Kumamoto, Japan; 7 Department of Pharmaceutical Microbiology, Graduate School of Pharmacological Sciences, Kumamoto University, Kumamoto, Japan; 8 Division of Developmental Genetics, IRDA, Kumamoto University, Kumamoto, Japan; 9 Department of Histology, Graduate School of Medical Sciences, Kumamoto University, Kumamoto, Japan

**Keywords:** Cardiac aging, Heart failure, Mitochondrial biogenesis, Calorie restriction

## Abstract

**Objective:**

Cardiac function declines with age, impairing exercise tolerance and negatively impacting healthy aging. However, mechanisms driving age-related declines in cardiac function are not fully understood.

**Methods:**

We examined mechanisms underlying age-related cardiac dysfunction using 3- and 24-month-old wild-type mice fed ad libitum or 24-month-old wild-type mice subjected to 70% calorie restriction (CR) starting at 2-month-old. In addition, cardiac aging phenotypes and mitochondrial biogenesis were also analyzed in 25-month-old cardiac-specific Hint1 knockout mice, 24-month-old CAG-Caren Tg mice, and 24-month-old wild-type mice injected with AAV6-Caren.

**Results:**

We observed inactivation of mitochondrial biogenesis in hearts of aged mice. We also showed that activity of the BAF chromatin remodeling complex is repressed by HINT1, whose expression in heart increases with age, leading to decreased transcription of Tfam, which promotes mitochondrial biogenesis. Interestingly, CR not only suppressed age-related declines in cardiac function and mitochondrial biogenesis but blocked concomitant increases in cardiac HINT1 protein levels and maintained Tfam transcription. Furthermore, expression of the lncRNA Caren, which inhibits Hint1 mRNA translation, decreased with age in heart, and CR suppressed this effect. Finally, decreased HINT1 expression due to Caren overexpression antagonized age-related declines in mitochondrial biogenesis, ameliorating age-related cardiac dysfunction, exercise intolerance, and exercise-induced cardiac damage and subsequent death of mice.

**Conclusion:**

Our findings suggest that mitochondrial biogenesis in cardiomyocytes decreases with age and could underlie cardiac dysfunction, and that the Caren-HINT1-mitochondrial biogenesis axis may constitute a mechanism linking CR to resistance to cardiac aging. We also show that ameliorating declines in mitochondrial biogenesis in cardiomyocytes could counteract age-related declines in cardiac function, and that this strategy may improve exercise tolerance and extend so-called "healthy life span".

## Highlights

• Increased HINT1 in aged hearts is linked to decreased *Tfam* expression.
• HINT1 decreases *Tfam* transcription by suppressing BAF chromatin remodeling complex.
• The expression of lncRNA *Caren* decreases with aging in heart.
• CR maintains *Caren* expression in heart, thereby preserving declines in mitochondria.
• Overexpression of *Caren* mitigates age-related declines in cardiac function.

## Graphical abstract

**Figure:**
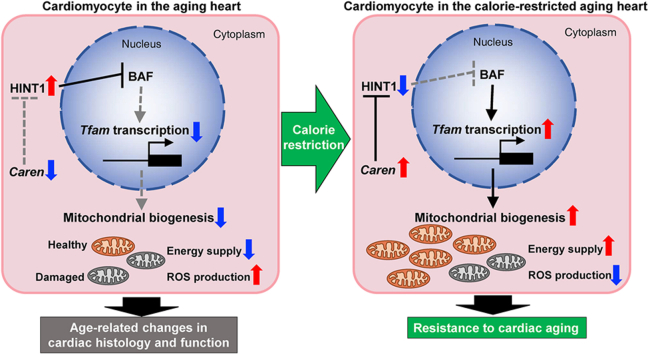

## 1 Introduction

As life expectancy increases worldwide, many countries are rapidly transitioning to aging societies. However, there is an approximately 10-year gap between healthy life expectancy, defined as the period during which a person can live without physical disability, and marginal life expectancy. Closing this gap is a global concern [1,2] and requires identification of factors that both decrease and increase healthy life expectancy.

For bodily organs to function normally throughout life, it is essential that their cellular components be supplied with sufficient oxygen and nutrients. Because the circulatory system transports oxygen and nutrients to organs throughout the body, circulatory insufficiency due to cardiovascular abnormalities, including cardiac dysfunction, is a major cause of age-related functional decline in various organs [3,4]. Therefore, it is critical to maintain normal heart function throughout life. According, we recently showed that elderly people, including centenarians, show a decline in activity and die within a few years of an episode of cardiac dysfunction characterized by high circulating NT-proBNP levels [5].

Even in non-elderly individuals with normal exercise capacity and healthy muscle and bone function, development of heart failure (HF) limits activity due to exercise intolerance [[6], [7], [8]]. Accordingly, ameliorating these declines should improve exercise tolerance in the elderly and increase healthy life expectancy and maximum life expectancy [[9], [10], [11]]. However, mechanisms driving such declines in cardiac function in the absence of organic heart disease, such as ischemic heart disease or heart valve disease, are not fully understood because cardiomyocytes, which are the parenchymal cells of the heart, do not proliferate or undergo cellular senescence, generally defined by cell cycle arrest [12,13]. Maintaining an adequate energy supply is especially important for cardiomyocytes to remain functional throughout an organism's lifespan. Mitochondria provide cells with ATP as an energy source, and mitochondrial dysfunction reportedly induces production of reactive oxygen species (ROS), increasing DNA damage and inflammation and leading to organ dysfunction [[14], [15], [16], [17], [18]].

Here, we found that aging mice exhibit pathological remodeling of cardiac tissue, including cardiomyocyte hypertrophy and cardiac fibrosis, conditions associated with decreased cardiac pump function and exercise tolerance, even in the absence of ischemic heart disease or valve heart disease. We also found that cardiac dysfunction seen in aging mice is due to decreases in mitochondrial mass resulting from inactivated mitochondrial biogenesis. Calorie restriction (CR) reportedly activates mitochondrial biogenesis and promotes resistance to age-related dysfunction in various organs [16,[19], [20], [21]]. Here we confirm those phenotypes and show that CR suppresses age-related increases in levels of Histidine triad nucleotide binding protein1 (HINT1) and maintains expression of mitochondrial transcription factor A (*Tfam*) in mouse heart. Relevant to mechanism, we observed that HINT1 suppressed chromatin remodeling activity of the BAF complex at the *Tfam* promoter, decreasing *Tfam* expression and impairing mitochondrial biogenesis. CR also suppressed age-related downregulation of the long non-coding RNA (lncRNA) *Caren*, which inhibits *Hint1* mRNA translation [22], in heart. Similar to CR, overexpressing *Caren* to block increases in HINT1 expression in mice activated mitochondrial biogenesis and antagonized age-related cardiac dysfunction and exercise intolerance. Overall, these findings suggest that mitochondrial biogenesis in cardiomyocytes decreases with age and could underlie cardiac dysfunction, and that the *Caren*-HINT1-mitochondrial biogenesis axis may constitute a mechanism linking CR to resistance to cardiac aging.

## 2 Results

### 2.1 Cardiac function declines with age in humans and mice

To assess age-associated changes in cardiac function in humans, we evaluated 28,260 male and female adults aged 18 years and older who underwent echocardiography as outpatients at the Department of Cardiovascular Medicine at Saga University Hospital between January 2014 and November 2022 (Supplementary Figures 1 and 2). Patients underwent echocardiography for several reasons, including preoperative cardiac function testing and diagnosis or follow-up of cardiovascular conditions such as ischemic heart disease, arrhythmias, cardiomyopathy and valvular heart disease. As shown in Supplementary Fig. 2, left ventricular contractility, estimated from left ventricular ejection fraction (LVEF), decreased mildly with age. Moreover, left ventricular diastolic dysfunction, estimated based on the ratios of early diastolic transmitral flow velocity (E) to late diastolic transmitral flow velocity (A) (E/A) and of E to early diastolic mitral annular velocity (e′) (E/e'), decreased markedly with age in parallel with increased left ventricular wall thickness, trends that showed no gender differences (Supplementary Fig. 2).

**Figure 1:**
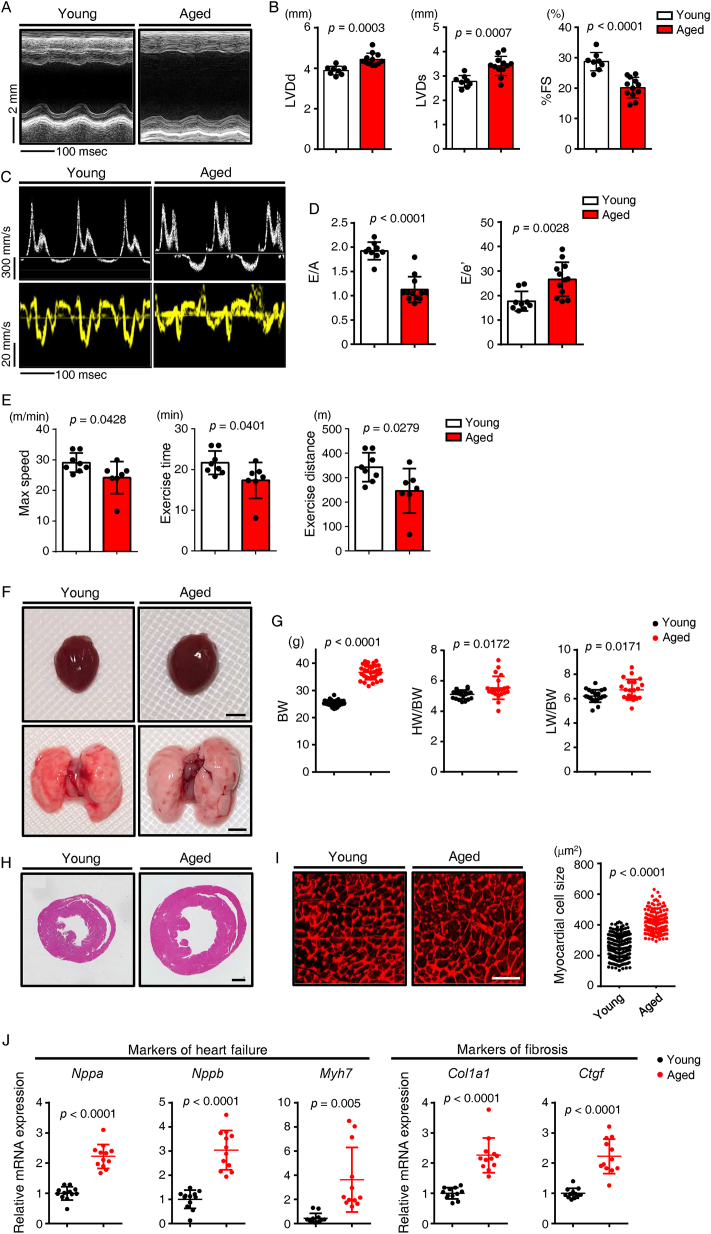
**Aged mice exhibit impaired cardiac function**. (A) Representative M-mode echocardiography recordings made in Young (3-months-old) and Aged (24-months-old) C57BL/6NJcl wildtype (WT) mice. (B) Left ventricular end-diastolic diameter (LVD; d) (mm) (left), left ventricular end-systolic diameter (LVD; s) (mm) (middle) and percent fractional shortening (%FS) (right) in indicated mice (*n* = 8, Young (3-month-old); n = 47, Aged (24-month-old)). (C) Representative transmitral flow velocity patterns by echocardiography for early transmitral flow velocity (E), atrial systolic velocity (A) (top row) and early diastolic mitral annular velocity (e′) (bottom row). (D) E/A (left) and E/e′ (right) ratios (*n* = 9, Young (3-month-old); n = 12, Aged (24-month-old)). (E) Results of treadmill test. Shown are maximal speed (left), total exercise time (middle), and exercise distance (right) (*n* = 8, Young; n = 7, Aged). (F) Gross appearance of whole heart (top row; scale bar, 5 mm) and whole lung (bottom row; scale bar, 5 mm) in indicated groups. (G) Body weight (BW) (g) (left), and HW/BW (mg g^−1^) (middle) and lung weight/body weight (LW/BW) (mg g^−1^) (right) ratios in indicated mice (*n* = 32 per group). (H) Hematoxylin-eosin (HE)-stained sections through the mid-portion of the heart. Scale bar, 1 mm. (I) Representative sections of left ventricle stained with wheat germ agglutinin (WGA) to indicate cardiomyocyte size in Young (3-months-old) and Aged (24-months-old) groups (left). Size distribution of myocardial cells (μm^2^) in indicated mice (right); number of cells = 283 (Young) and 277 (Aged). Scale bar, 100 μm. (J) Relative expression of genes associated with heart failure and fibrosis in hearts of indicated mice (*n* = 9, Young; n = 12, Aged). Expression levels in 3-month-old mice were set to 1. Statistical significance was determined by a two-sided unpaired Student's *t*-test (B, D, E, G, I, and J).

Next, to evaluate comparable effects in mice, we assessed cardiac function and performed histological analysis of 3-month-old (young) versus 24-month-old (aged) male mice. Echocardiography analysis revealed that, compared to young mice, aged mice developed cardiac hypertrophy with moderate left ventricular dilatation and showed impaired cardiac systolic function, as estimated by fractional shortening (Figure 1A,B). Cardiac diastolic function also became impaired with age (Figure 1C,D). Treadmill testing also revealed significantly reduced exercise tolerance in aged compared to young mice (Figure 1E). Heart weight/body weight (HW/BW) and lung weight/body weight (LW/BW) ratios increased in aged relative to young mice (Figure 1F,G). Moreover, cardiomyocyte enlargement, determined based on histological analysis of heart tissue, was significantly greater in aged relative to young mice (Figure 1H,I). RT-PCR analysis of heart tissue showed that expression of markers of HF (*Nppa*, *Nppb*, and *Myh7*) and cardiac fibrosis (*Col1a1* and *Ctgf*) significantly increased in aged relative to young mice (Figure 1J). Overall, based on analysis of male mice, we conclude that cardiac systolic and diastolic function decline with age in parallel with development of cardiomyocyte hypertrophy and fibrosis, similar to outcomes seen in human heart [22,23].

### 2.2 Mitochondrial biogenesis decreases in the aging mouse heart

**Figure 2:**
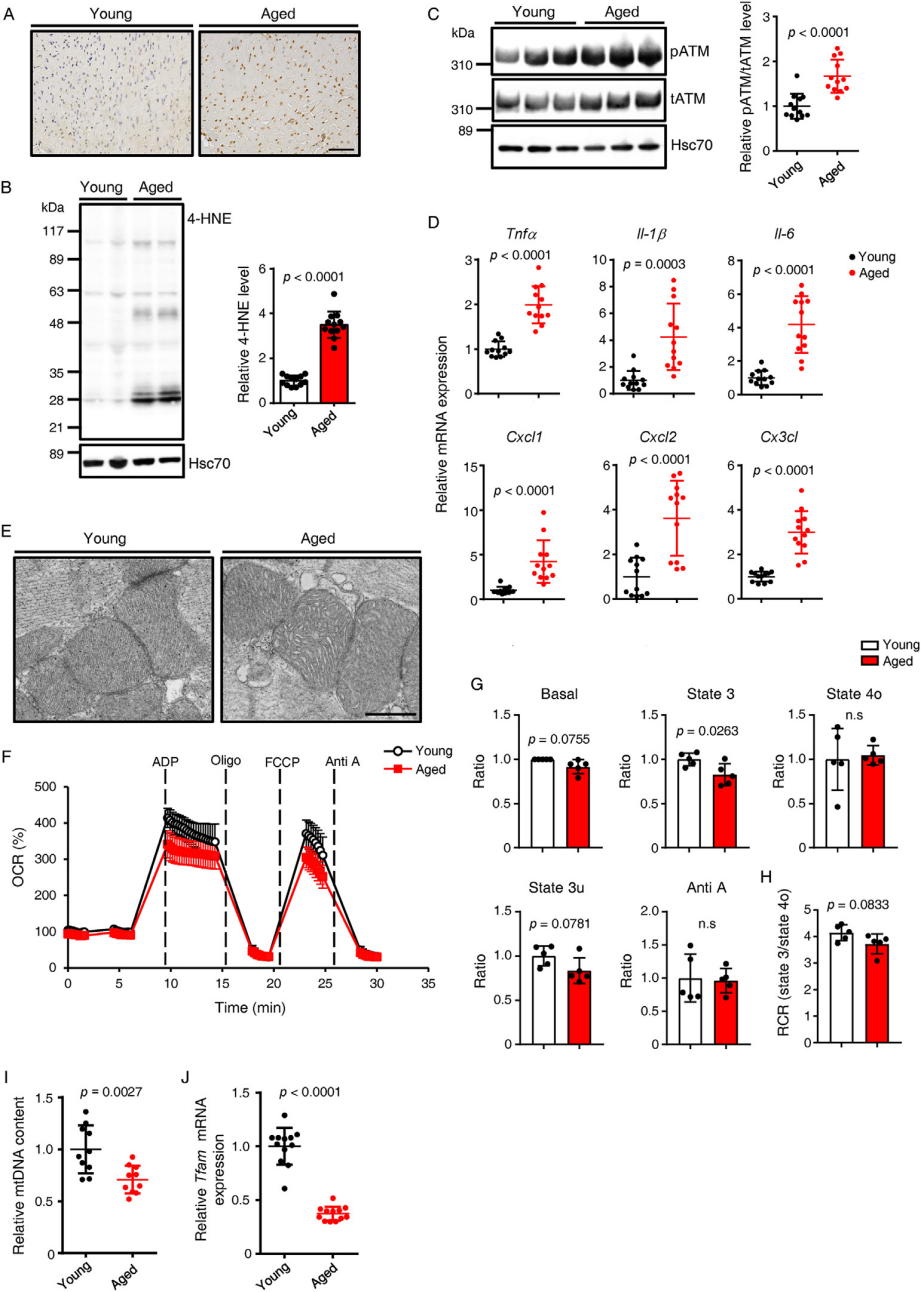
**Hearts of aged mice show DDR activation and altered mitochondrial homeostasis**. (A) Immunohistochemical analysis of representative sections of mouse heart tissues stained with 8-OHdG, a marker of oxidative DNA damage. Young, 3-months-old; Aged, 24-months-old. Scale bar, 100 μm. (B and C) Representative western blots (left) and corresponding quantitation (right) in heart tissues of indicated mice probed with antibodies to (B) 4-hydroxy-2-nonenal (4-HNE), an indicator of lipid-derived oxidative stress product, and (C) pATM/total ATM (*n* = 12 per group). Hsc70 served as loading control. Values in Young groups were set to 1. (D) Relative expression of genes associated with inflammation in hearts of indicated mice. Values seen in Young mice were set at 1 (*n* = 10 per group). (E) Representative electron micrographs of heart tissues from indicated mice (scale bar, 5 μm). (F) Oxygen consumption rate (OCR) in mitochondria isolated from heart tissues of indicated mice (n = 5 per group). Basal values seen in Young mice were set at 100%. (G) Quantification of basal respiration, ATP-linked respiration in the presence of ADP (State 3), resting respiration in the presence of oligomycin (State 4o), maximal uncoupling respiration in the presence of FCCP (State 3u), and respiration in the presence of antimycin A (Anti A) in indicated mice (n = 5 per group). Values in young mice were set at 1. (H) Respiratory control ratio (RCR, state 3/state 4o) of mitochondria isolated from hearts of indicated mice (*n* = 5 per group). (I) Analysis of mitochondrial DNA (mtDNA) content (*Cytb*/*ActB*) in heart tissues of indicated groups (*n* = 12 per group). WT values were set to 1. (J) Relative *Tfam* mRNA expression in heart tissues of indicated mice (*n* = 10 per group). Statistical significance was determined by a two-sided unpaired Student's *t*-test (B toD and G toJ). n.s, not significant, between groups.

To define mechanisms underlying age-related declines in cardiac function, we compared relevant activities in hearts of aged versus young mice. Immunostaining of heart tissue for 8-hydroxy-2′-deoxyguanosine (8-OHdG) (Figure 2A) and western blotting for 4-hydroxynonenal (4-HNE) (Figure 2B) revealed enhanced ROS accumulation in hearts of aged (24-month-old) versus young (3-month-old) mice. Moreover, levels of phosphorylated ataxia telangiectasia mutated (ATM) protein, an important regulator of the DNA damage response (DDR), significantly increased in hearts of aged mice (Figure 2C), potentially in response to ROS-induced DNA damage. Consistent with reports that DDR activation initiates an inflammatory cascade [22,24,25], we observed significantly increased levels of transcripts related to inflammation in hearts of aged mice (Figure 2D). Since mitochondria play a key role in ROS production [25], we performed electron microscopy (EM) analysis of mitochondrial structure in heart and found that aged mice showed altered mitochondrial cristae structure relative to that seen in young mice (Figure 2E). Moreover, oxygen consumption rate (OCR) in mitochondria isolated from hearts of aged mice were decreased compared to rates seen in young mice (Figure 2F–H), indicating impaired mitochondrial respiratory function. Furthermore, mitochondrial DNA (mtDNA) content was significantly lower in hearts of aged versus young mice (Figure 2I). TFAM binds mtDNA to regulate mitochondrial biogenesis [26]. We observed significantly decreased *Tfam* expression in hearts of aged versus young mice (Figure 2J), suggesting that *Tfam* downregulation may underlie age-related decreases in mitochondrial biogenesis and mitochondrial mass.

### 2.3 Calorie restriction (CR) antagonizes phenotypes associated with cardiac aging and blocks age-related increases in HINT1 expression

**Figure 3:**
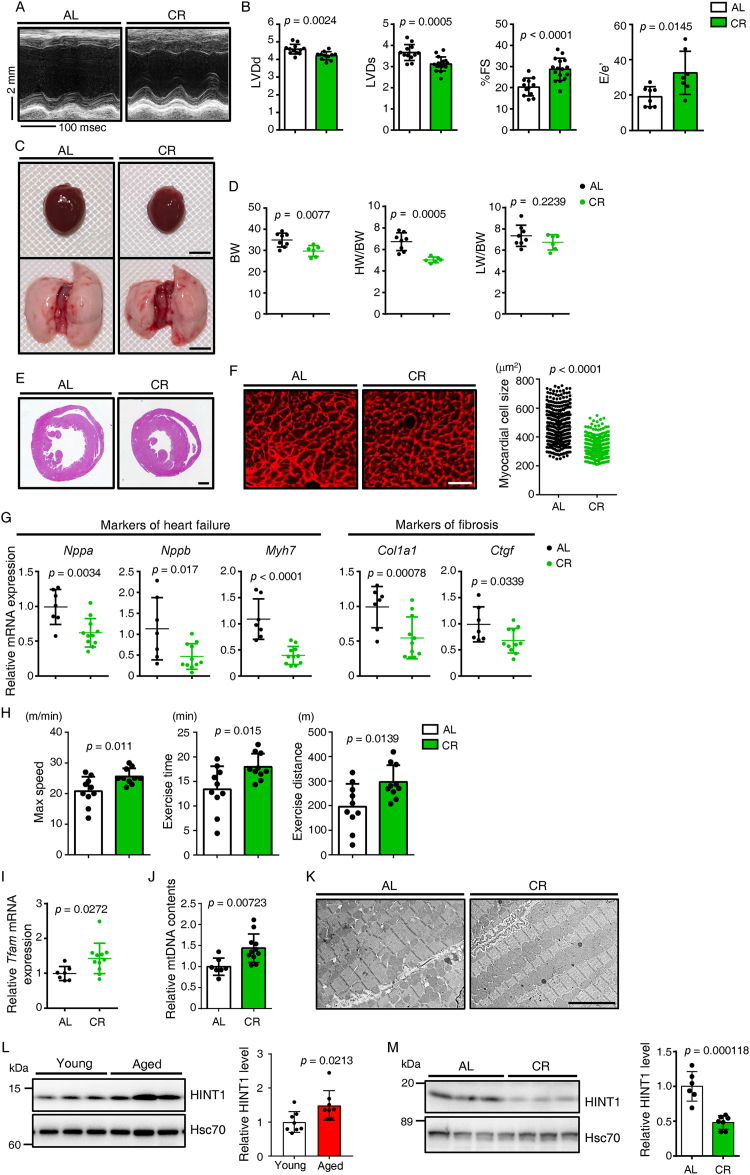
**Calorie restriction attenuates cardiac aging phenotypes and age-related increases in HINT1 expression**. (A) Representative M-mode echocardiography recordings made in 24-month-old C57BL/6NJcl mice fed ad libitum (AL) or subjected to 70% Carorie restriction (CR). (B) Shown are echocardiographic measurements including left ventricular end-diastolic diameter (LVD; d) (mm) (left), left ventricular end-systolic diameter (LVD; s) (mm) (middle), percent fractional shortening (%FS) (right) and early transmitral flow velocity (E) to early diastolic mitral annular velocity (e′) ratios (E/e′) in indicated mice (*n* = 8, AL; *n* = 7, CR). (C) Gross appearance of whole heart (top row; scale bar, 5 mm) and whole lung (bottom row; scale bar, 5 mm) in indicated groups. (D) Body weight (BW) (g) (left) and HW/BW (mg g^−1^) (middle) and lung weight/body weight (LW/BW) (mg g^−1^) (right) ratios in indicated mice (*n* = 7, AL; *n* = 6, CR). (E) Hematoxylin-eosin (HE)-stained sections through the mid-portion of the heart. Scale bar, 1 mm. (F) Representative sections of left ventricle stained with wheat germ agglutinin (WGA) to indicate cardiomyocyte size in AL and CR groups (left). Size distribution of myocardial cells (μm^2^) in indicated mice (right); number of cells = 355 per group. Scale bar, 100 μm. (G) Relative expression of genes associated with heart failure and fibrosis in hearts of indicated mice (*n* = 7, AL; *n* = 11, CR). Expression levels in the AL group were set to 1. (H) Treadmill analysis showing maximal speed (left), total exercise time (middle), and exercise distance (right) of indicated groups (*n* = 10, AL; *n* = 10, CR). (I) Relative *Tfam* mRNA expression in heart tissue of indicated mice (*n* = 7, AL; *n* = 11, CR). Expression levels in the AL group were set to 1. (J) Analysis of mitochondrial DNA (mtDNA) content (*Cytb*/*ActB*) in heart tissues of indicated groups (*n* = 7, AL; *n* = 11, CR). AL values were set to 1. (K) Representative electron micrographs of heart tissue from indicated groups (scale bar, 5 μm). (L and M) Representative immunoblotting (left) and quantification (right) of HINT1 protein levels in (L) heart tissues of young (3-month-old) and aged (24-month-old) mice (*n* = 7 per group) or (M) heart tissues of AL (*n* = 6) and CR (*n* = 7) groups. Hsc70 served as loading control. Values in Young or AL group were set to 1. Statistical significance was determined by a two-sided unpaired Student's *t*-test (B, D, F, G, H to J, L, and M).

CR, which is reportedly the most effective strategy to extend both lifespan and healthy lifespan across species [21], activates mitochondrial biogenesis and ameliorates age-related dysfunction in multiple organs [16,[19], [20], [21]]. Indeed, echocardiographic analysis of 24-month-old male CR mice subjected to 70% CR starting at 2-months-old revealed that, relative to comparably-aged mice fed ad libitum (AL), both cardiac hypertrophy and left ventricular dilatation were attenuated, with preserved systolic and diastolic function (Figure 3A,B). Age-related increases in HW/BW ratio were significantly suppressed in CR relative to AL mice (Figure 3C,D) as was cardiomyocyte enlargement (Figure 3E,F). RT-PCR analysis of heart tissue showed significantly decreased levels of transcripts associated with HF, cardiac fibrosis, and inflammation in CR compared to AL mice (Figure 3G and Supplementary Fig. 3A). Moreover, treadmill testing showed that CR significantly improved exercise tolerance in aged mice (Figure 3H). In addition, transcript levels in heart of several genes encoding factors functioning in mitochondrial biogenesis, including *Tfam*, significantly increased in CR versus AL mice (Figure 3I and Supplementary Fig. 3B), as did mtDNA content (Figure 3J). Moreover, based on EM analysis hearts of CR mice exhibited a greater number of intermyofibrillar mitochondria relative to AL mice (Figure 3K). These analyses confirm that CR counteracts cardiac aging phenotypes and age-related declines in mitochondrial biogenesis in heart.

### 2.4 HINT1 negatively regulates *Tfam* transcription

**Figure 4:**
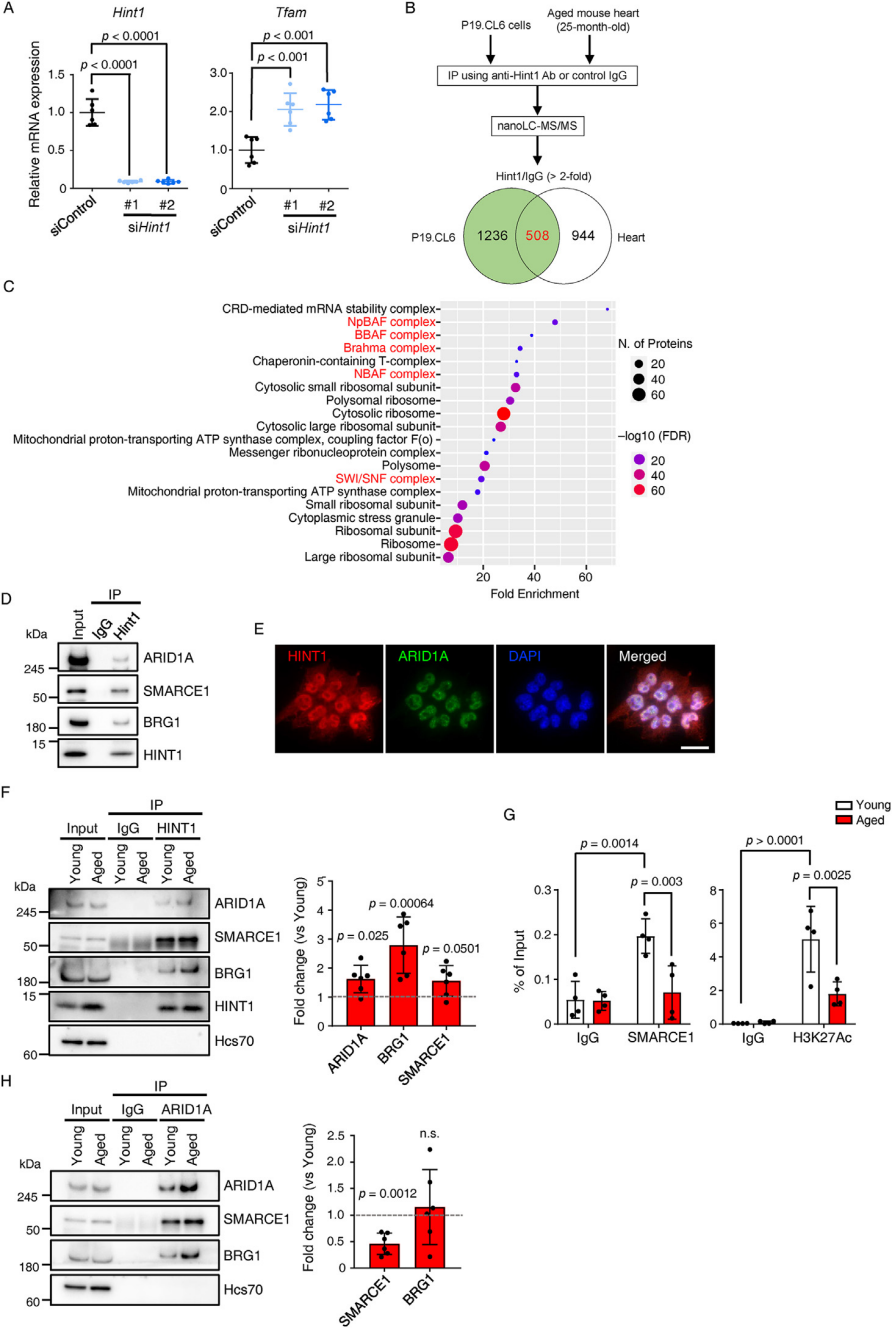
**HINT1 contributes to age-related *Tfam* downregulation in heart**. (A) Relative expression of *Hint1* and *Tfam* in P19.CL6 cells transfected with negative control siRNA (siControl) or *Hint1* siRNAs (si*Hint1*-A and si*Hint1*-B) (*n* = 6 per group). Values in control group were set to 1. (B) Schematic diagram showing IP coupled to mass spectrometry (IP-MS) analysis and Venn diagram showing the number of identified HINT1-interacting protein candidates in P19.CL6 cells and heart tissue of aged mice (24-months-old), or candidate proteins overlapping between P19.CL6 cells and heart tissue. (C) Gene ontology (GO) enrichment analysis of cellular compartment of the 508 overlapping HINT1-interacting protein candidates. Shown is a bubble plot representing top 20 enriched terms sorted by the average of their ranks based on both false discovery rate (FDR) and fold-enrichment. (D) IP of HINT1 protein in P19.CL6 cells. Shown is representative immunoblotting for ARID1A, SMARCE1, BRG1, and HINT1. (E) Representative immunofluorescence staining for HINT1 (red) and ARID1A (green) in P19.CL6 cells. Nuclei were counterstained with DAPI (blue). Scale bar, 20 μm. (F) IP of HINT1 protein in heart tissues of young (3-months-old) and aged (24–26-months-old) mice. Shown is representative immunoblotting for ARID1A, SMARCE1, BRG1, and HINT1 (left). Hsc70 served as loading control. Levels of indicated proteins co-IP'd with HINT1 in hearts of aged relative to young mice (right, *n* = 6). (G) CUT&RUN analysis of SMARCE1 and H3K27Ac in heart tissues of young (3-months-old) and aged (24–26-months-old) mice (n = 4). (H) IP of ARID1A protein in heart tissues of young (3-months-old) and aged (24–26-months-old) mice. Shown is representative immunoblotting for ARID1A, SMARCE1, and BRG1 (left). Hsc70 served as loading control. Levels of indicated proteins co-IP'd with ARID1A in hearts of aged relative to young mice (right, *n* = 6). Statistical significance was determined by a two-sided unpaired Student's *t*-test (F, H) or one-way ANOVA followed by Sidak's *post hoc* test (A, G). n.s, not significant, between groups. (For interpretation of the references to color in this figure legend, the reader is referred to the Web version of this article).

We previously reported that expression of HINT1 increases in hearts of pressure overload-induced HF model mice and that HINT1 expression is upregulated in heart tissue from HF patients [22]. HINT1 also regulates transcription of genes associated with mitochondrial biogenesis [27,28]. Interestingly, we found that cardiac HINT1 protein levels increase with age in mice (Figure 3L) and humans (Supplementary Fig. 4). Moreover, CR suppressed age-related increases in HINT1 protein levels in mouse heart (Figure 3M), suggesting that these effects may underlie CR-dependent activation of mitochondrial biogenesis. To determine whether HINT1 negatively regulates *Tfam* expression, we knocked down (KD) HINT1 in mouse embryonal carcinoma P19.CL6 cells and observed upregulated *Tfam* expression (Figure 4A). HINT1 reportedly regulates activity of Microphthalmia transcription factor (MITF), which induces expression of *peroxisome proliferator-activated receptor gamma coactivator 1 alpha* (*Pgc1α*), in turn activating *Tfam* transcription [28]. HINT1 binds to MITF in the cytoplasm, inhibiting its nuclear translocation and function [27]. Wnt-β-catenin signaling also regulates *Pgc1α* and *Nrf1* transcription [29], and HINT1 suppresses β-catenin-mediated transcription by interacting with the transcriptional repressors Pontin and Reptin [30]. Thus, we asked whether HINT1 downregulates *Tfam* expression by interacting with either MITF, Pontin, or β-catenin proteins. However, immunoprecipitation (IP) analysis indicated that HINT1 did not interact with these proteins (Supplementary Fig. 5), suggesting that HINT1 downregulates *Tfam* in heart via other mechanisms.

### 2.5 HINT1 suppresses BAF complex-mediated *Tfam* transcription in the aged heart

To identify mechanisms underlying *Tfam* suppression by HINT1 in heart, we used IP coupled to mass spectrometry (IP-MS) to identify HINT1-interacting proteins. Specifically, we IP'd HINT1 proteins from either whole lysates of P19.CL6 cells or heart tissue of aged mice, and identified co-IP'd proteins by quantitative proteomic analysis with nanoscale liquid chromatography with tandem mass spectrometry (nanoLC-MS/MS) (Figure 4B and Supplementary Table 1). Overall, we identified 1744 proteins from P19.CL6 cells and 1452 from heart tissue as candidate HINT1-interactors (anti-HINT1 antibody-IP versus control IgG-IP ratio >2). Among these, 508 overlapped between P19.CL6 cells and heart tissue. Moreover, gene ontology (GO) enrichment analysis of those proteins revealed that among the top 20 terms, five were associated with the BAF chromatin remodeling complex (also known as SWI/SNF) [31,32] (Figure 4C and Supplementary Table 2). Also, co-IP analysis of P19.CL6 cells showed HINT1 interaction with AT-rich interactive domain-containing protein 1 (ARID1A), SWI/SNF-related matrix associated actin-dependent regulator of chromatin E1 (SMARCE1), and BRM/SWI2-related gene-1 (BRG1), all BAF complex components [31,32] (Figure 4D). Finally, immunofluorescence analysis showed nuclear colocalization of HINT1 and ARID1A (Figure 4E). These results suggest overall that HINT1 interacts with the BAF complex.

Since cardiac HINT1 protein levels increase with age, we examined HINT1 interactions with various BAF complex proteins in hearts of young and aged mice by co-IP analysis (Figure 4F). We observed HINT1 interactions with complex components ARID1A, SMARCE1, and BRG1 in young and aged mice, but quantitative analysis indicated an increase in those interactions in aged mice (Figure 4F). BAF complex activity reportedly facilitates binding of transcriptional activators or repressors to gene regulatory regions [31,32]. Thus, we asked whether increased HINT1/BAF complex interaction alters BAF complex binding to the *Tfam* promoter region in hearts of young and aged mice using the CUT&RUN protocol. That analysis revealed that *Tfam* promoter occupancy by SMARCE1 decreases in aged relative to young mice (Figure 4G). Furthermore, acetylation of histone H3 lysine 27 (H3K27Ac), an active promoter/enhancer mark, decreased in aged relative to young mice (Figure 4G). Finally, levels of co-IP'd SMARCE1/ARID1A significantly decreased in hearts of aged compared to young mice (Figure 4H), suggesting that HINT1 binding impairs BAF complex integrity and that HINT1 may interact with SMARCE1. To test this possibility, we conducted co-IP analysis using *Smarce1* KO P19.CL6 cells (Supplementary Fig. 6A). We observed that SMARCE1-deficiency did not alter HIINT1 interaction with ARID1A and BRG1, suggesting that HINT1 interacts with BAF complex components other than SMARCE1. These findings suggest overall that HINT1 interaction decreases binding of the BAF complex to the *Tfam* promoter, decreasing *Tfam* expression.

To further investigate BAF complex contributions to *Tfam* expression, we examined *Tfam* expression in the mouse myoblast line C2C12 treated with ACBI1, a proteolysis targeting chimera (PROTAC) degrader of BAF complex ATPases [33] essential for chromatin remodeling, such as BRG1 (Supplementary Fig. 6B). *Tfam* expression showed a small but significant decrease in ACBI1-treated compared with untreated control (DMSO) cells (Supplementary Fig. 6C). However, ACBI1-treated and untreated cells showed comparable mtDNA content (Supplementary Fig. 6D). ACBI1-treated cells showed significantly decreased expression levels of genes encoding transcription factors functioning in mitochondrial biogenesis, such as *Nrf1* and *Nrf2*, relative to untreated cells. However, expression levels of the coactivator *Pgc1α*, an essential activator of *Tfam* transcription [26], were comparable in ACBI1-treated and untreated cells (Supplementary Fig. 6C). On the other hand, in addition to significant *Tfam* downregulation and reduced mtDNA content (Figure 2I,J), expression levels of *Pgc1α*, *Nrf1*, and *Nrf2* significantly decreased in hearts of aged compared to young mice (Supplementary Fig. 6E). These results suggest that in the aging heart, inhibition of BAF complex-mediated chromatin remodeling, together with *Pgc1α* downregulation, significantly decreases *Tfam* transcript levels, decreasing mitochondrial biogenesis. Thus, BAF complex inhibition is necessary for *Tfam* downregulation but most likely insufficient by itself to decrease mitochondrial biogenesis.

### 2.6 Blocking increases in HINT1 expression activates mitochondrial biogenesis and antagonizes age-related cardiac phenotypes

Above we showed increased HINT1 protein in hearts of aging humans and mice (Figure 3L and Supplementary Fig. 4) and decreased HINT1 protein in hearts of CR mice (Figure 3M). Thus, we hypothesized that like CR, blocking increases in HINT1 expression in the aging heart may promote resistance to cardiac aging. To test this possibility, we generated cardiac-specific *Hint1* knockout (*Hint1*^*Flox*/*Flox*^; *αMHC*-*Cre*, CKO) mice (Supplementary Fig. 7) and examined age-related cardiac phenotypes in 25-month-old males CKO and littermate controls (*Hint1*^*Flox*/*Flox*^). HINT1 protein levels in hearts of aged CKO mice were significantly lower than those seen in aged control mice (Supplementary Fig. 7C). Echocardiographic analysis of aged CKO mice revealed attenuation of age-related declines in both cardiac contractility and diastolic function relative to aged control mice (Supplementary Figs. 8A–D). In addition, age-related increases in the HW/BW ratio were also blocked in aged CKO relative to control mice (Supplementary Figs. 8E and F). Moreover, cardiomyocyte enlargement, determined based on histological analysis of heart tissue, was significantly greater in aged relative to young mice (Supplementary Figs. 8G and H). RT-PCR analysis also revealed that age-related increases in expression of HF and cardiac fibrosis markers in heart tissue were suppressed in aged CKO mice (Supplementary Fig. 8I). Furthermore, expression of genes associated with mitochondrial biogenesis, including *Tfam*, significantly increased in hearts of aged CKO relative to control mice (Supplementary Fig. 8J), and aged CKO mice also showed significantly increased mtDNA content in heart tissue (Supplementary Fig. 8K). Taken together, these results suggest that blocking HINT1 expression in cardiomyocytes counteracts age-related decline in mitochondrial biogenesis and cardiac function.

**Figure 5:**
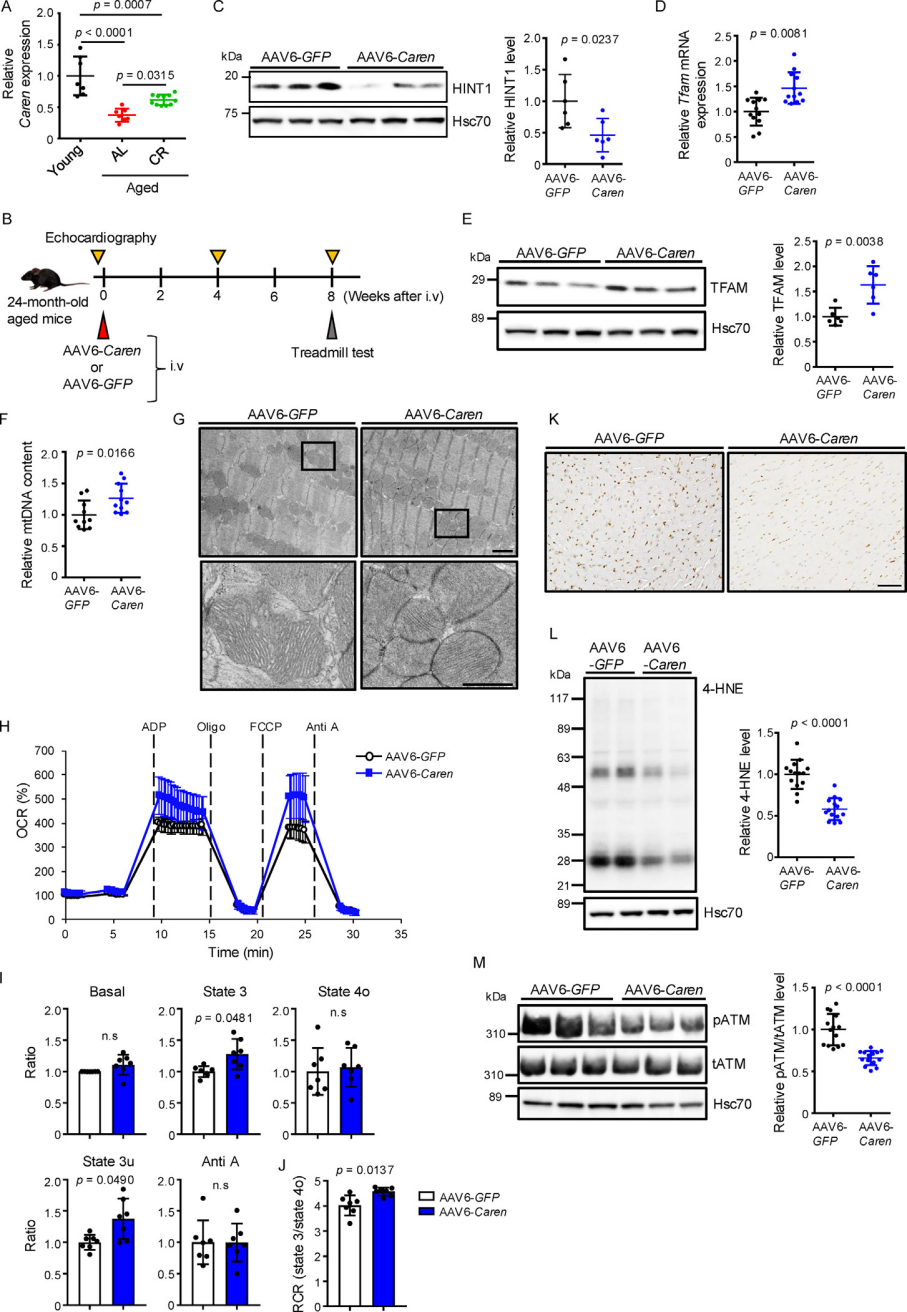
**AAV6-*Caren* injection rescues impaired mitochondrial biogenesis and function seen in aged mice**. (A) Relative *Caren* expression in hearts of Young (3-month-old) mice and Aged (24-month-old) mice fed ad libitum (AL) or subjected to 70% calorie restriction (CR) (*n* = 7, Young and AL; *n* = 11, CR). Values in the Young group were set to 1. (B) Design of experimental analysis of AAV6-*Caren*-injected aged mice. (C) Representative immunoblotting (left) and quantification (right) of HINT1 proteins in heart tissues of AAV6-*GFP*- or AAV6-*Caren*-treated mice (*n* = 6 per group). Hsc70 served as loading control. Values in the AAV6-*GFP* group were set to 1. (D–F) Relative *Tfam* mRNA expression (D), representative western blots (left) and quantitation (right) of TFAM protein levels (E), and mtDNA content (*Cytb*/*ActB*) (F) in heart tissues of indicated mice (*n* = 12 per group). (G) (top row) Representative electron micrographs of heart tissue from indicated groups (scale bar, 5 μm). (bottom row) Higher magnification views of corresponding boxed areas shown above (scale bar, 1 μm). (H) OCR analysis of mitochondria isolated from heart tissues of indicated groups (n = 7 per group). Basal OCR values in the AAV6-*GFP* group were set at 100%. (I) Quantification of basal respiration, ATP-linked respiration in the presence of ADP (State 3), resting respiration in the presence of oligomycin (State 4o), maximal uncoupling respiration in the presence of FCCP (State 3u), and respiration in the presence of antimycin A (Anti A) (n = 7 per group) in indicated groups. Values in the AAV6-*GFP* group were set at 1. (J) Respiratory control ratio (RCR, state 3/state 4o) of mitochondria isolated from hearts of mice in indicated groups (n = 7 per group). (K) Immunohistochemical 8-OHdG staining of heart tissue from control AAV6-*GFP*- or AAV6-*Caren*-treated mice. Scale bar, 1 mm. (L) Representative western blots (left) and quantitation (right) of staining for 4-HNE-modified proteins in heart tissues of indicated groups (*n* = 14 per group). (M) Representative western blots (left) and quantitation of pATM/total ATM protein levels (right) in heart tissues of indicated groups (*n* = 14 per group). Hsc70 served as loading control. Values in the AAV6-*GFP* group were set to 1. Statistical significance was determined by a two-sided unpaired Student's *t*-test (C to F, I, J, L, and M) or one-way ANOVA followed by Tukey's *post hoc* test (A). n.s, not significant, between groups.

We previously reported that *Caren* counteracts HF development by interacting with *Hint1* mRNA and blocking its translation via various intermediate(s)—among them, an RNA binding protein [22]. Interestingly, we observed significantly decreased levels of *Caren* transcripts in hearts of aged compared to young mice and that CR suppressed those decreases (Figure 5A). Thus, we asked whether decreasing HINT1 expression via overexpressing *Caren* in the aged heart would activate mitochondrial biogenesis and prevent age-related cardiac changes. To do so, first assessed 24-month-old transgenic (Tg) mice overexpressing *Caren* under control of the CAG promoter (CAG-*Caren* Tg mice) [22] versus WT littermate controls, and observed significantly reduced HINT1 protein levels in heart tissues of Tg versus WT mice (Supplementary Fig. 9A). Transcript levels of several genes encoding factors involved in mitochondrial biogenesis, including *Tfam*, were significantly increased in hearts of CAG-*Caren* Tg versus WT mice (Supplementary Fig. 9B), as were TFAM protein levels (Supplementary Fig. 9C) and mtDNA content (Supplementary Fig. 9D). Moreover, echocardiographic analysis of 24-month-old Tg mice revealed that, relative to WT controls, both cardiac hypertrophy and left ventricular dilatation were attenuated, with preserved systolic and diastolic function (Supplementary Figs. 10A–D). Age-related increases in HW/BW ratio were significantly suppressed in CAG-*Caren* Tg relative to WT mice (Supplementary Figs. 10E and F), as was cardiomyocyte enlargement in heart, based on histological analysis (Supplementary Figs. 10G and H). RT-PCR analysis of heart tissue showed significantly decreased levels of transcripts associated with HF and cardiac fibrosis in *Caren* Tg versus WT controls (Supplementary Fig. 10I). ROS accumulation and upregulation of inflammatory genes were also significantly blocked in heart tissue of Tg relative to WT mice (Supplementary Figs. 9E–G). Furthermore, aged *Caren* Tg mice exhibited increased exercise tolerance (Supplementary Fig. 10J).

We next asked whether increasing myocardial mitochondrial biogenesis in aging mice would ameliorate age-related cardiac dysfunction and underlying myocardial changes. To do so, we injected 24-month-old mice exhibiting age-related cardiac dysfunction intravenously with 1 × 10^11^ vg per mouse recombinant adeno-associated virus serotype 6 (AAV6)-*Caren* to restore *Caren* transcripts in cardiomyocytes, and injected controls with 1 × 10^11^ vg per mouse of recombinant AAV6-green fluorescent protein (GFP) (Figure 5B). Eight weeks later, immunoblotting analysis indicated significantly decreased HINT1 protein levels in hearts of AAV6-*Caren* relative to control mice (Figure 5C). AAV6-*Caren* treatment also significantly increased *Tfam* mRNA and protein levels and mtDNA content in heart tissue relative to control levels (Figure 5D–F). EM analysis revealed a greater number of intermyofibrillar mitochondria exhibiting fine cristae structure, which is typically seen in hearts of young mice (Figure 2E), in hearts of aged AAV6-*Caren* relative to control mice (Figure 5G). OCR of mitochondria isolated from hearts of aged AAV6-*Caren* mice also improved compared to controls (Figure 5H–J), although ROS accumulation and DDR activation in heart tissue decreased relative to that seen in controls (Figure 5K–M).

**Figure 6:**
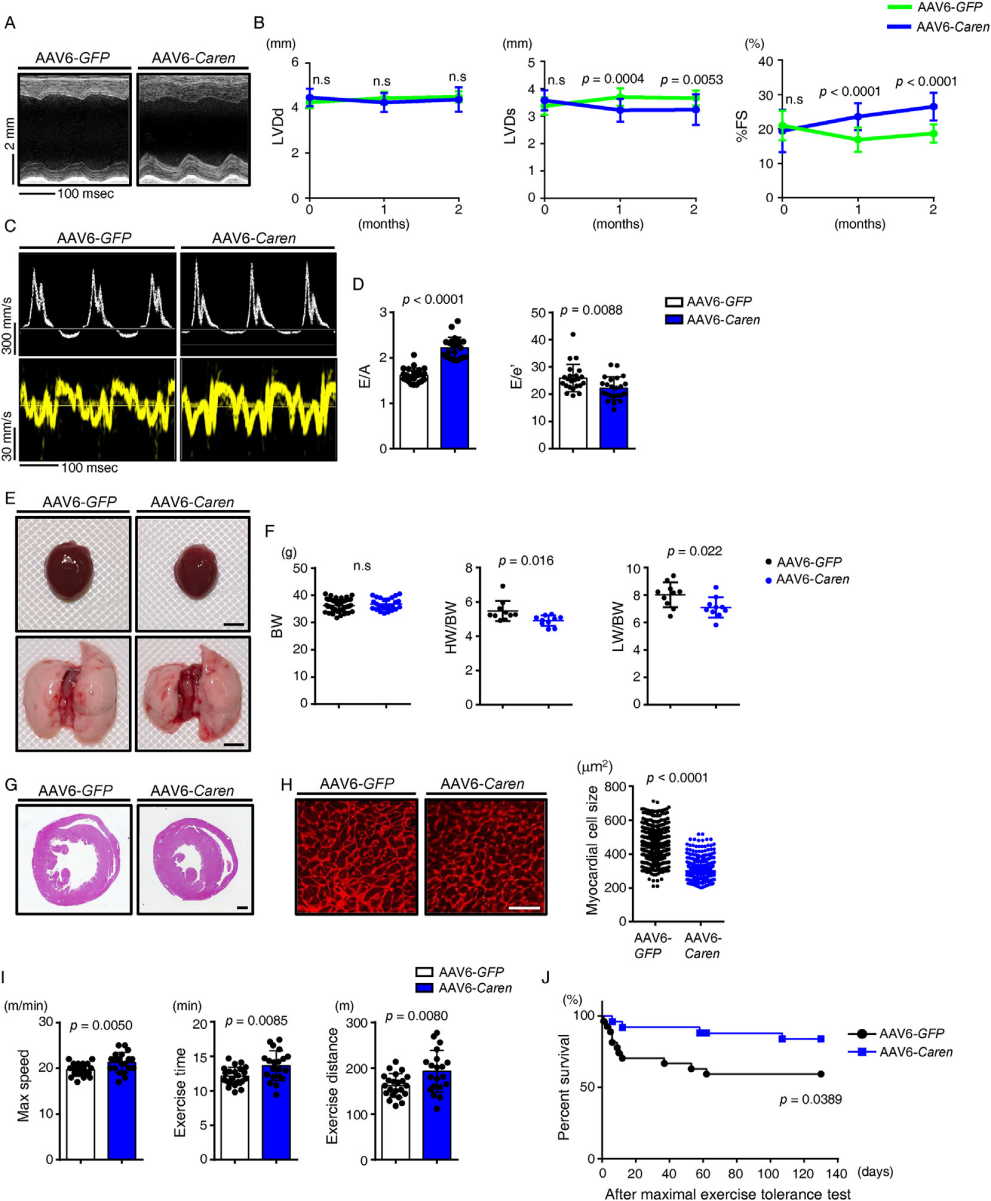
**AAV6-*Caren* injection rescues age-related cardiac dysfunction and exercise intolerance**. (A) Representative M-mode echocardiography recordings in indicated mice. (B) Left ventricular end-diastolic diameter (LVD; d) (mm) (left), left ventricular end-systolic diameter (LVD; s) (mm) (middle) and percent fractional shortening (%FS) (right) at time 0 and at 1 and 2 months after injection of AAV6 *Caren* or control *GFP* (*n* = 30 per group). (C) Representative transmitral flow velocity patterns by echocardiography for early transmitral flow velocity (E), atrial systolic velocity (A) (top row) and early diastolic mitral annular velocity (e′) (bottom row) in indicated groups. (D) E/A (left) and E/e′ (right) ratios (*n* = 23 per group). (E) Gross appearance of whole heart (top row; scale bar, 5 mm) and whole lung (bottom row; scale bar, 5 mm). (F) Body weight (BW) (g) (left), and HW/BW (mg g^−1^) (middle) and lung weight per body weight (LW/BW) (mg g^−1^) (right) ratios in indicated mice (*n* = 10 per group). (G) Hematoxylin-eosin (HE) staining of sections through the mid-portion of the heart in indicated groups (scale bar, 1 mm). (H) Representative sections of left ventricle stained with WGA to indicate cardiomyocyte size (left; scale bar, 100 μm) and size distribution (*n* = 654 cells per group) of myocardial cells (μm^2^) (right) in indicated mice. (I) Treadmill analysis showing maximal speed (left), total exercise time (middle), and exercise distance (right) of indicated groups (*n* = 22, AAV6-*GFP*; *n* = 20, AAV6-*Caren*). (J) Proportion of mice surviving after the maximal exercise intolerance test in indicated groups (n = 27, AAV6-*GFP*; *n* = 25, AAV6-*Caren*). Statistical significance was determined by a two-sided unpaired Student's *t*-test (B, D, F, H, and I) or log lank test (J). n.s, not significant, between groups.

Blood pressure and heart rate were comparable in AAV6-*Caren* and control mice (Supplementary Fig. 11A). Interestingly, echocardiographic analysis showed that age-related progression of cardiac left ventricular dilatation, systolic dysfunction and diastolic dysfunction was ameliorated in AAV6-*Caren*-treated compared to control mice (Figure 5, Figure 6A–D). In addition, cardiac hypertrophy, as assessed by HW/BW ratio and histological analysis, and cardiac function, as assessed by pulmonary congestion based on LW/BW ratio, were improved in AAV6-*Caren* relative to control mice (Figure 6E–H). RT-PCR analysis of heart tissue showed that upregulation of genes associated with HF and cardiac fibrosis was blocked in AAV6-*Caren* relative to control mice (Supplementary Fig. 11B). Moreover, treadmill testing showed that AAV6-*Caren* treatment significantly improved exercise tolerance in aged mice, which showed little or no post-exercise immobility due to breathlessness and decreased mortality after maximal exercise than controls (Figure 6I,J, Supplementary Movie 1 and 2). Collectively, these results suggest that *Caren*-mediated inhibition of age-related increases in cardiac HINT1 expression activates mitochondrial biogenesis by enhancing *Tfam* expression and improves cardiac function, outcomes that may counteract exercise intolerance and exercise-related mortality, both of which increase with age.

Supplementary video related to this article can be found at https://doi.org/10.1016/j.molmet.2025.102107

The following are the supplementary data related to this article:

VideoS1 **Endurance exercise testing of AAV6-*Caren*-treated aged mice.**
Movie of aged WT mice injected with AAV6-*Caren* or AAV6-*GFP* taken during endurance exercise testing.4 VideoS1

VideoS2 **Behavior of AAV6-*Caren*-treated aged mice after endurance exercise testing.**
Movie of aged WT mice injected with AAV6-*Caren* or AAV6-*GFP* taken immediately after endurance exercise testing.5 VideoS2

## 3 Discussion

In this study, we found that even in the absence of valvular or ischemic heart disease, aged mice show pathological remodeling of cardiac tissue, such as cardiomyocyte hypertrophy, cardiac fibrosis, and accompanying decreases in cardiac pump function and exercise tolerance. These age-related changes were associated with decreased mitochondrial biogenesis in mouse cardiomyocytes. We also show that decreases in mitochondrial biogenesis due to decreased *Tfam* transcription likely occur via inactivation of the BAF complex resulting from increased HINT1 levels in the aging heart. Expression of *Caren*, which blocks *Hint1* mRNA translation, decreased in the aging heart. Interestingly, CR antagonized many of these age-related declines and preserved exercise tolerance. Moreover, CR suppressed increased HINT1 expression by maintaining *Caren* expression and maintained *Tfam* expression, thereby activating mitochondrial biogenesis. Finally, we confirmed that overexpressing *Caren* blocked increases in HINT1 expression and restored activation of mitochondrial biogenesis, counteracting age-related cardiac changes, improving exercise intolerance and reducing exercise-induced cardiac damage and subsequent death of mice. These findings support the idea that the *Caren*-HINT1-mitochondrial biogenesis axis is an important mechanism linking CR to resistance to cardiac aging.

It was previously reported that CR activates sirtuin and AMP-activated protein kinase (AMPK) pathways, increasing PGC1α expression and activation [[19], [20], [21]]. PGC1α together with NRF1/2 induces expression of genes encoding mitochondrial proteins, such as TFAM, and enhances mitochondrial biogenesis [26]. Indeed, CR mice showed increased cardiac *Pgc1α*, *Nrf1*, and *Nrf2* expression relative to AL mice (Supplementary Fig. 3B). Interestingly, we also demonstrated that expression of these genes in hearts of aged CAG-*Caren* Tg mice was significantly higher than that in comparably-aged WT controls (Supplementary Fig. 9B). These findings support our conclusion that *Caren*-mediated pathways are closely related to CR-mediated activation of mitochondrial biogenesis.

The BAF complex regulates transcription via chromatin remodeling activity [31,32]. We observed both decreased SMARCE1 occupancy and decreased H3K27Ac levels at the *Tfam* promoter in hearts of aged versus young mice. Thus, in addition to increasing *Pgc1α* and *Nrf1/2* expression in heart, *Caren* may promote BAF-mediated epigenetic changes at the *Tfam* regulatory region by suppressing HINT1 expression, thereby activating mitochondrial biogenesis. *Caren-*overexpressing mice also showed increased exercise tolerance and decreased incidence of exercise-induced acute heart failure and death, suggesting that activating mitochondrial biogenesis is could serve as an effective strategy to maintain myocardial mitochondrial mass in the aging heart.

We also observed a decline in cardiac function in hearts of aging mice due to decreases in mitochondrial mass, which we attribute to inactivated mitochondrial biogenesis. Similar age-related decreases in mitochondrial mass were evident in kidney, liver, and muscle (Supplementary Fig. 12), suggesting that these activities underlie functional declines in various organs. Currently, mechanisms underlying these outcomes are not fully understood, although it was recently reported that mice show increased mitophagy activity with age in various organs [34]. Mitochondrial mass is determined by the balance between mitochondrial biogenesis and mitophagy [26]. Moreover, CR reportedly activates not only mitochondrial biogenesis but also mitophagy in various organs [[19], [20], [21]]. Therefore, it is now important to assess multiple organs in aging animals to determine how CR alters this balance in a manner that promotes resistance to aging.

We found that *Caren* transcript levels in heart decreased with age, an activity suppressed by CR. Mechanisms underlying regulation of *Caren* transcripts in heart remain unclear. lncRNA transcript levels are regulated by both transcription and miRNA- and RNA binding protein-mediated stabilization or degradation of lncRNA [35]. Future studies should address which of those mechanisms govern *Caren* transcript levels in the aging heart.

We demonstrated that HINT1 interacts with the BAF complex via interaction with complex components other than SMARCE1. However, the HINT1 binding motif required for BAF complex interaction remains unclear. HINT1 exhibits nucleotide phosphoramidase activity and zinc- and calmodulin-regulated small ubiquitin-like modifier (SUMO) protease activity, and the HINT1 calmodulin binding motif and histidine triad are essential for these activities and for substrate binding [36,37]. Moreover, HINT1 reportedly interacts with Pontin and Reptin via its N-terminal region [30]. These HINT1 motifs may contribute to HINT1 interaction with the BAF complex. Further studies are required to identify both the relevant HINT1 binding motif and the HINT1 binding partner among BAF complex components.

The decline in cardiac function seen in aged mice was very mild compared to the pathophysiology of HF but was associated with a decline in physical activity, as exemplified by exercise tolerance analysis. This finding suggests that since it is critical for the elderly to maintain physical activity in order to live a healthy lifestyle, it is important to prevent or ameliorate age-related declines in cardiac function.

In conclusion, this study demonstrates that perturbation of mitochondrial homeostasis with age is associated with age-related pathological remodeling of cardiac tissue and cardiac dysfunction. Specifically, in aging hearts, increased HINT1 expression decreases mitochondrial biogenesis by suppressing *Tfam* transcription, and CR likely antagonizes these phenotypes by blocking increases in HINT1 expression via maintenance of *Caren* expression. We also show that ameliorating declines in mitochondrial biogenesis in cardiomyocytes could counteract age-related declines in cardiac function, and that this strategy may improve exercise tolerance and extend so-called “healthy life span”.

## 4 Materials and methods

### 4.1 Animals

All experimental procedures were approved by the Ethics Review Committee for Animal Experimentation at Kumamoto University, Kumamoto, Japan (approval No. A27-063, A29-072, A2019-063, A2021-071, and A2023-039). Procedures followed the guidelines and recommendations outlined by the NIH Guide for the Care and Use of Laboratory Animals (8th Edition, 2011). Prior to tissue collection, mice were euthanized under isoflurane anesthesia, followed by cervical dislocation to ensure rapid euthanasia, a procedure also employed during sacrifice for tissue harvesting. Euthanasia was performed when animals met the established criteria for humane endpoints, specifically a body weight loss exceeding 20% over a 3-day period or 25% over a 1-week period, in accordance with the Kumamoto University Ethics Review Committee for Animal Experimentation above.

All animals were fed a normal diet (CE-2, CLEA, Tokyo, Japan), bred in a mouse house with automatically controlled lighting (12 h on, 12 h off), and maintained at a stable temperature of 22 ± 2 °C and a relative humidity of 40–80%.

C57BL/6NJcl (3- and 24-month-old) WT male mice were used in this study. Genetically engineered mice used in this study were: Tg mice overexpressing *Caren* driven by the CAG promoter (CAG-*Caren*) [22] on a C57BL/6N background. To maintain an isogenic strain, CAG-*Caren* Tg mice were propagated as hemizygotes by breeding with C57BL/6N mice.

### 4.2 Calorie restriction

Two-month-old C57BL/6NJcl male mice were randomly assigned to ad libitum (AL) and calorie restriction (CR) groups. Both groups were fed normal chow (CE-2, CLEA). To determine baseline ad libitum levels per day, food intake in the AL group was measured once every week. In the CR group, mice were fed 70% of food consumed by the AL group three times a week during daytime periods.

### 4.3 Generation of *Hint1* conditional KO mice

Mutagenized sperm from mice on a C57BL/6N background (EM:05220) was purchased from the European Mouse Mutant Archive (EMMA, http://www.infrafrontier.eu/) and used for *in vitro* fertilization with C57BL/6N eggs. To generate *Hint1* conditional KO mice (*Hint1*^*Flox*/*Flox*^) through Flp/FRT-mediated recombination, pronuclear embryos were electroporated with Flp mRNA (800 ng μl^−1^) using the Super Electroporator NEPA 21 (NEPA GENE, Chiba, Japan). Genotyping was performed by PCR of tail DNA using the following primers: *Hint1*cKO-S (5′-GTCTATGAGTGGATTTGTTACTCTGG-3′) and *Hint1*cKO-AS (5′-TTATTAAAGACCACTTAAACTACC-3′). To maintain the strain, mice were propagated as heterozygotes by breeding with C57BL/6N mice. Tg mice overexpressing *Cre* driven by the murine *αMHC* promoter (*αMHC*-*Cre*) [38] were kindly provided by Prof. Kinya Otsu (National Cerebral and Cardiovascular Center, Japan).

### 4.4 Human research participants

The study was approved by the Ethics Committees of Kumamoto University (approval No. 2671) and Saga University (approval No. 2022-10-SCR-05). A total of 70,417 patients enrolled at the Department of Cardiovascular Medicine, Saga University Hospital, between January 2014 and November 2022 and underwent echocardiography. Among those cases, only data from the first echocardiography was analyzed for patients who had undergone more than one echocardiography during this period. Thus, the final analysis included data from 28,260 patients (Supplementary Fig. 1).

For immunohistochemical analysis of human heart tissue samples from atrium biopsy cases (Supplementary Fig. 4), the study was approved by the Ethics Committees of Kumamoto University (approval No. 2640 and 2641) and Saga University (approval No. 2020-01-01 and 2020-09-01).

### 4.5 Echocardiography in mice

Mice were preconditioned by chest hair removal with a topical depilatory (FUJIFILM VisualSonics, Toronto, Canada), anaesthetized with 1.5–2.5% isoflurane administered via inhalation, and maintained in a supine position on a dedicated animal handling platform with limbs immobilized for electrocardiogram gating during imaging. Body temperature was kept constant by relaying signals from a rectal probe to a heating pad, while heart and respiratory rates were continuously monitored. Transthoracic echocardiography was performed using a high frequency ultrasound system dedicated to small animal imaging (VisualSonics Vevo 3100, FUJIFILM VisualSonics, Toronto, Canada) using a MS 400 linear array transducer (18–38 MHz). M-mode recording was performed at the midventricular level. All images were analyzed using dedicated software (Vevo Labo version 5.7.1). LV wall thickness and internal cavity diameters at diastole (LVID; d) and systole (LVID; s) were measured. Per cent LV fractional shortening (%FS) was calculated from M-mode measurements. For analysis of cardiac diastolic function, transmitral flow velocity patterns and early diastolic mitral annular velocity (e′) were measured to calculate early transmitral flow velocity (E) to atrial systolic velocity (A) (E/A) and ratio E to e′ (E/e′) in 4-chamber view. All procedures were performed under double-blind conditions with regard to genotype or treatment.

### 4.6 Mouse endurance exercise testing

Experimental mice were allowed to adapt to the treadmill chamber (Model MK-690S/4M, Muromachi, Japan) for 15 min with unlimited movement 3 times on separate days. After confirming that mice were sufficiently accustomed to running, we began treadmill testing at 5 m/min, and after 30 s, increased speed to 6 m/min. After the first minute, speed was increased first to 8 m/min and then to 10 m/min after another minute. Speed was then increased by 1 m/min every minute.

The experiment was terminated when a mouse touched the electrodes on ten or more occasions during rest intervals. Electrodes were positioned at the rear end of the treadmill belt.

### 4.7 RT-PCR analysis

Total RNA was extracted using a RNeasy Mini Kit (Qiagen, Valencia, CA, USA). DNase-treated RNA was reverse-transcribed using a Prime Script RT reagent Kit (Takara Bio Inc, Shiga, Japan). Heart tissue was homogenized using a multi-beads shocker (Yasui Kikai, Osaka, Japan). Real-time quantitative RT-PCR was performed using TB Green Premix Ex Taq II (Takara Bio Inc), and a Thermal Cycler Dice Real-Time system (Takara Bio Inc). Relative transcript abundance was normalized to that of *18S* rRNA levels in mouse and human samples. Primer sets used for RT-PCR are listed in Supplementary Table 3.

### 4.8 Quantification of mitochondrial DNA content

To assess mitochondrial DNA (mtDNA) content, we determined mtDNA/nuclear DNA (nDNA) ratios by extracting DNA from mouse tissues and subjecting it to real-time quantitative PCR analysis using specific primer sets for mtDNA (*Cytb* and *Nd2*) and nDNA (*ActB* and *B2m*) (Supplementary Table 4). Real-time quantitative PCR was performed using TB Green Premix Ex Taq II (Takara Bio Inc) and a Thermal Cycler Dice Real-Time system (Takara Bio Inc).

### 4.9 Western blotting

Mouse heart tissue was homogenized in lysis buffer (10 mM Tris–HCl, 1% Triton X-100, 50 mM NaCl, 30 mM sodium pyrophosphate, 50 mM NaF, 5 mM EDTA, 0.1 mM Na_3_VO_4_, plus a protease inhibitor cocktail (Nacalai Tesque, Kyoto, Japan), pH 7.5) using a multi-beads shocker (Yasui Kikai). Proteins (20 μg) were separated by SDS–PAGE and transferred to PVDF membranes. Membranes were incubated with anti-4-hydroxy-2-nonenal (4-HNE) (clone HNEJ-2, MHN, Japan Institute for the Control of Aging (JaICA), NIKKEN SEIL, Shizuoka, Japan), anti-phosphorylated ATM (Ser1981) (10H11.E12, #4526, Cell Signaling Technology), anti-ATM (D2E2, #2873, Cell Signaling Technology), anti-TFAM (ab131607, Abcam, Cambridge, UK), anti-HINT1 (ab124912, Abcam), anti-ARID1A (D2A8U, #12354S, Cell Signaling Technology), anti-BRG1 (D1Q7F, #49360S, Cell Signaling Technology), or anti-SMARCE1 (E6H5J, #33360S, Cell Signaling Technology). Antibodies were diluted 1:1,000, and samples were incubated at 4 °C overnight. After TBST washing, membranes were incubated with 1:2,000 diluted horseradish peroxidase (HRP)-conjugated donkey anti-rabbit IgG or sheep anti-mouse IgG (GE Healthcare Life Science, Piscataway, NJ, USA) antibodies at room temperature for 60 min. Internal controls were incubated with 1:2,000 diluted anti-Hsc70 (sc-7298, Santa Cruz Biotechnology, Santa Cruz, CA, USA) antibody, and 1:2,000 diluted HRP-conjugated sheep anti-mouse IgG (GE Healthcare Life Science) antibody was used as a secondary antibody.

In some experiments, mouse anti-rabbit IgG (Conformation Specific) mAb (HRP-conjugated) (L27A9, #5127, Cell Signaling Technology) was used as a secondary antibody to avoid masking signals from proteins of interest by signals from antibodies used in the IP.

Immunodetection was carried out using ECL Western Blotting Detection Reagents or ECL Prime Western Blotting Detection Reagents (both from GE Healthcare Life Science).

### 4.10 Cell culture and transfection

The mouse embryonal carcinoma cell line P19.CL6 (RIKEN BRC, Tsukuba, Japan) was maintained in MEMα supplemented with 10% fetal calf serum (FCS) under 5% CO2 and 95% air. The human embryonic kidney line HEK293 and the mouse myoblast line C2C12 (ATCC, Manassas, VA, USA) were maintained in DMEM supplemented with 10% FCS under 5% CO2 and 95% air.

P19.CL6 cells were transfected with Mission siRNA Universal Negative Control (SIC001, Sigma–Aldrich, St Louis, MO, USA) or mouse *Hint1*-targeting siRNA (si*Hint1*-#1: SASI_Mm01_00048024: GCAUAUAUCCCAGAUUUCUTT, si*Hint1*-#2: SASI_Mm01_00048025: GGUACCGGAUGGUGGUGAATT, Sigma–Aldrich) using Lipofectamine RNAi MAX reagent (Thermo Fisher Scientific Inc.) according to the manufacturer's instructions. After transfection, cells were subjected to RT-PCR analyses.

HEK293 cells were transfected with an expression plasmid encoding Flag-tagged *Hint1* or HA-tagged *Mitf* alone or co-transfected with both vectors using Lipofectamine 3000 reagent (Thermo Fisher Scientific Inc.) according to the manufacturer's instructions. After transfection, cells were harvested and subjected to IP analysis.

C2C12 cells were incubated 72 h with 1 μM ACBI1(Selleck Biotech, Tokyo, Japan). Cells were then harvested and subjected to RT-PCR analysis, western blotting, or quantification of mtDNA content.

### 4.11 Establishment of *Smarce1* knockout cell lines

*Smarce1* knockout and control lines were established using a Guide-it CRISPR/Cas9 system (Takara Bio Inc) according to the manufacturer's instructions. In brief, sense (CCGGTTGATTCTCCTACCGTGACC) and antisense (AAACGGTCACGGTAGGAGAATCAA) oligos corresponding to mouse *Smarce1*-targeting sgRNA were annealed and cloned into the pGuide-it ZsGreen1 vector (Takara Bio Inc). To construct the control sgRNA plasmid, Guide-it control annealed oligos (Takara Bio Inc) were cloned into the vector. P19. CL6 cells were transfected with plasmids harboring either *Smarce1-*targeting or control sgRNA using the *Trans*IT-X2 Dynamic Delivery System (Mirus Bio, Madison, WI, USA) according to the manufacturer's instructions and cultured for 48 h. Cells were harvested and ZsGreen1-positive cells were isolated using a cell sorter SH800S (SONY, Tokyo, Japan). Established cell clones were subjected to immunoblotting analysis to confirm *Smarce1* knockout.

### 4.12 Immunoprecipitation

Cultured cells were washed with PBS and homogenized in 1% Tx-100 lysis buffer (20 mM Tris–HCl, 150 mM NaCl, 1 mM EDTA, 1% Triton X-100, 10 mM NaF, 2 mM Na_3_VO_4_, 10 mM Na_4_P_2_O_7_, 10% glycerol, plus cOmplete, EDTA-free protease inhibitor cocktail (Roche, Mannheim, Germany), pH 7.5). Mouse heart tissue was homogenized in 1% Tx-100 lysis buffer using a multi-beads shocker (Yasui Kikai). Samples were incubated 10 min on ice and centrifuged. Supernatants served as protein extracts, which were subjected to IP.

For co-IP of HINT1 and ARID1A, 0.6–1.0 mg of protein was incubated with 4 μg of anti-HINT1 antibody (#10717-1-AP, Proteintech Japan Inc., Tokyo, Japan), anti-ARID1A antibody (#12354S, Cell Signaling Technology), or control normal rabbit IgG (#2729, Cell Signaling Technology) overnight at 4 °C. Samples were incubated 2 h with Dynabeads Protein G (Thermo Fisher Scientific Inc.) at 4 °C. After washing with 1 % Tx-100 lysis buffer, IP'd proteins were eluted with 1 × SDS sample buffer (50 mM Tris–HCl, 2% SDS, 0.1% 2-mercaptoehanol, 10% glycerol, and bromophenol blue, pH 6.8), and purified proteins were immunoblotted.

To calculate fold-change of a protein co-IP'd with HINT1 or ARID1A in hearts of aged and young mice, we employed the following formula (where x = HINT1 or ARID1A and y = BRG1 or SMARCE1):

$$\text{Fold}\;\text{change}\;\left(\text{vs}\;\text{Young}\right)=\frac{\text{Aged}\;\left[\;\frac{\text{IP}\;\left(y\right)\;/\;\left(\text{Input}\;\left(y\right)\;/\;\text{Hsc}70\right)}{\text{IP}\;\left(x\right)\;/\;\left(\text{Input}\;\left(x\right)\;/\;\text{Hsc}70\right)}\right]}{\text{Young}\;\left[\;\frac{\text{IP}\;\left(y\right)\;/\;\left(\text{Input}\;\left(y\right)\;/\;\text{Hsc}70\right)}{\text{IP}\;\left(x\right)\;/\;\left(\text{Input}\;\left(x\right)\;/\;\text{Hsc}70\right)}\right]}$$

For co-IP of Flag-tagged HINT1, 1.0 mg protein was incubated 2 h with 30 μl of Anti-DDDDK-tag mAb-Magnetic Beads (MBL) at 4 °C. After washing with 1% Tx-100 lysis buffer, IP'd proteins were eluted with 1 × SDS sample buffer, and purified proteins were immunoblotted.

To identify proteins interacting with HINT1 in P19.CL6 cells or mouse heart tissue, IP coupled to mass spectrometry (IP-MS) analysis was conducted using anti-HINT1 antibody (#10717-1-AP, Proteintech Japan Inc.) or control normal rabbit IgG (#2729, Cell Signaling Technology). After IP, precipitates were washed with 1% Tx-100 lysis buffer and then subjected to quantitative proteomic analysis.

### 4.13 Quantitative proteomic analysis

Peptide samples for quantitative proteomics were prepared by trypsin digestion using SP3 method [39]. Analysis was conducted as described [40]. Briefly, each sample was analyzed by data-independent acquisition (DIA/SWATH) on the TripleTOF 6600 (SCIEX, Framingham, MA, USA) interfaced with the Eksigent nanoLC 400 (SCIEX). Protein identification and quantification were conducted using the library-free search function in DIA-NN 1.8 with the UniProt mouse reference proteome allowing one miss-cleavage [41]. Concentration of each protein was calculated from specific peptides using the MaxLFQ algorithm [42], which was integrated into DIA-NN. Peptides and proteins were filtered at a false discovery rate (FDR) of <1% for identification and quantification.

To determine HINT1-interactors, proteins detected in IP-MS analysis using anti-HINT1 antibody or control IgG in P19.CL6 cells and aged mouse heart tissue were filtered at an anti-HINT1 antibody-IP versus control IgG-IP ratio of >2-fold. HINT1-interacting proteins found in both P19.CL6 cells and aged mouse heart tissue underwent gene ontology (GO) enrichment analysis using the graphical gene-set enrichment tool ShinyGO v0.80 [43]. Enriched GO terms were investigated and enriched terms (FDR <0.05) were sorted by the average of their ranks based on FDR and fold-enrichment. A bubble plot showing the top 20 enriched terms was generated using ShinyGO v0.80.

### 4.14 Immunofluorescence staining

Cells were fixed in 4% PFA at room temperature for 10 min. After washing with PBS, cells were incubated with PBS containing 0.2% Triton X-100 for 10 min and blocked with PBS containing 0.05% Tween 20, 0.1% BSA, and 5% normal goat serum. For double-staining with anti-HINT1 (#10717-1-AP, Proteintech Japan Inc.) and anti-ARID1A (#12354S, Cell Signaling Technology) antibodies, anti-ARIDA1 antibody was labeled with a fluorescent dye CoraLite Plus 488 using a FlexAble Antibody Labeling Kit (Proteintech Japan Inc.), according to the manufacturers' instructions. Cells were incubated overnight at 4 °C with anti-HINT1 antibody (1:200). After PBS/0.05% Tween 20 (PBST) washing, samples were incubated 1 h with Alexa Fluor 594-conjugated anti-rabbit IgG (1:400, Thermo Fisher Scientific Inc.) antibody at room temperature. After PBST washing, cells were incubated overnight at 4 °C with CoraLite Plus 488-labbeled anti-ARID1A antibody (1:100). After PBST washing, nuclei were counterstained with NucBlue Fixed Cell ReadyProbes Reagent (Thermo Fisher Scientific Inc.). Images were obtained using a BZ-X710 microscope (Keyence, Osaka, Japan).

### 4.15 CUT&RUN analysis

CUT&RUN analysis was carried out using a CUT&RUN Assay Kit (Cell Signaling Technology), according to the manufacturers' instructions. In brief, mouse heart tissues were fixed 10 min with 0.1% formaldehyde at room temperature and homogenized using a Dounce homogenizer. Tissue homogenates served as single-cell suspension, which was incubated with Concanavalin A magnetic beads. Cells immobilized on beads were then treated with digitonin to permeabilize them and then incubated 2 h with anti-SMARCE1 antibody (1:50, #33360S, Cell Signaling Technology), anti-H3K27Ac antibody (1:50, #8173S, Cell Signaling Technology), or control normal IgG (1:25, #66362, Cell Signaling Technology) at 4 °C. Samples were washed and incubated 1 h with pAG-MNase at 4 °C. After washing, MNase was activated by adding CaCl_2_ and samples were incubated 30 min at 4 °C. Samples were incubated 10 min at 37 °C to allow diffusion of digested chromatin fragments into the supernatant. DNA was purified from supernatants and subjected to real-time PCR analysis. Primer pairs used are shown in Supplementary Table 5. For normalization, Sample Normalization Spike-In yeast DNA was added to each sample and signals were quantified using primers for the *S. cerevisiae ACT1* gene.

### 4.16 Immunohistochemical analysis

Immunohistological analysis of 8-OHdG was performed as described [44]. Briefly, mouse heart tissue samples were fixed 24 h in 4% paraformaldehyde and embedded in paraffin. Blocks were sectioned into 4-μm-thick sections, air-dried, and deparaffinized. For immunohistochemistry, sections were pretreated with periodic acid (Nichirei Biosciences, Tokyo, Japan) to inhibit endogenous peroxidases. Specimens were then incubated overnight with anti-8-hydroxy-2′-deoxyguanosine (8-OHdG) (1:20, MOG, JaICA, NIKKEN SEIL). After PBS washing, sections were immunostained using a Histofine mouse stain kit (Nichirei Biosciences), according to the manufacturers' instructions, and specimens counterstained with hematoxylin. As negative controls isotype control IgG was used instead of primary antibodies. Peroxidase activity was visualized by incubation with a 3,3-diaminobenzidine solution. For wheat germ agglutinin (WGA) labeling, sections were incubated 1 h with 5 mg/ml Alexa Fluor 594-conjugated WGA (W11262, Invitrogen, Waltham, MA, USA) at room temperature. After washing, sections were cover-slipped with a water-soluble antifading mounting medium containing 4′,6-diamidino-2-phenylindole (DAPI) (Vibrance Antifade Mounting Medium with DAPI, H-1800, Vector Laboratories, Newark, CA, USA). Images were obtained using a BZ-X710 microscope (Keyence).

### 4.17 Transmission electron microscopy

Mouse heart tissue was fixed 4 h in phosphate buffer containing 2% paraformaldehyde and 2.5% glutaraldehyde (pH 7.4) at 4 °C. After washing in phosphate buffer at 4 °C, specimens were postfixed 2 h in 1% osmium tetroxide at 4 °C. Specimens were then washed repeatedly in distilled water, stained 30 min with 1% uranyl acetate, dehydrated through a graded ethanol series and propylene oxide, and embedded in Glicidether (Selva Feinbiochemica, Heidelberg, Germany). Ultrathin sections were cut and mounted onto nickel grids, stained 10 min with 1% uranyl acetate and 5 min with Reynolds lead citrate, and then observed using a HT7700 electron microscope (Hitachi High-Tech Corporation, Tokyo, Japan) [45].

### 4.18 AAV production and mouse treatment

To construct AAV plasmids encoding *Caren* or *GFP*, respective cDNAs were cloned into the pAAV-CMV vector (Takara Bio Inc). Recombinant AAV6 vectors were produced with an AAVpro Helper Free System (Takara Bio Inc) and purified using an AAVpro Purification Kit (Takara Bio Inc), according to the manufacturer's instructions. Genome copy number was determined using an AAVpro Titration Kit (for Real-time PCR) Ver. 2 (Takara Bio Inc). 24-month-old male C57BL/6NJcl mice were intravenously injected with AAV6-*Caren* or AAV6-*GFP* vectors at 1 × 10^11^ vg. Cardiac function was assessed by echocardiography before injection and at 4 and 8 weeks afterwards. After echocardiography, mice were sacrificed and heart tissues subjected to histological, RT-PCR, immunoblot, and oxygen consumption analyses.

### 4.19 Isolation of mouse heart mitochondria

Mouse heart tissue was homogenized in mitochondrial isolation buffer (210 mM mannitol, 70 mM sucrose, 5 mM HEPES-KOH, 1 mM EGTA, 0.5% fatty acid-free bovine serum albumin (BSA), pH 7.2) using a glass-teflon homogenizer. Homogenates were centrifuged at 27,000×*g* for 10 min at 4 °C, and pellets suspended in mitochondrial isolation buffer. Samples were centrifuged at 500×*g* for 5 min at 4 °C, and supernatants were collected and centrifuged at 10,000×*g*, 4 °C for 5 min. The pellet was then resuspended in mitochondrial isolation buffer, and samples centrifuged 5 min at 10,000×*g* at 4 °C. Resulting pellets were suspended in BSA-free mitochondrial isolation buffer, and mitochondrial protein concentration determined using the Bio-Rad Protein Assay Dye Reagent (Bio-Rad Laboratories, Hercules, CA, USA). Samples were then incubated with an equal volume of mitochondrial isolation buffer and subjected to oxygen consumption analysis.

### 4.20 Oxygen consumption analysis of isolated mitochondria

Isolated mitochondria were diluted with mitochondrial assay solution (220 mM mannitol, 70 mM sucrose, 2 mM HEPES-KOH, 1 mM EGTA, 10 mM KH_2_PO_4_, 5 mM MgCl_2_, 0.2% fatty acid-free BSA, 2 mM malate, 10 mM pyruvate, pH 7.2) to 80 μg/ml. Diluted mitochondria were loaded at 50 μl per well into 24-well Seahorse assay plates (Seahorse Bioscience) and centrifuged at 2,000×*g* for 20 min at 4 °C. After centrifugation, 450 μl mitochondrial assay solution was added to wells, and plates were incubated 8 min at 37 °C. To assess mitochondrial respiratory function, the following compounds (final concentrations) were sequentially-injected into each well: ADP (4 mM), oligomycin (2 μM), FCCP (4 μM), and Antimycin A (4 μM). OCR was measured under basal conditions and after each injection using an XFe24 extracellular flux analyzer (Seahorse Bioscience).

### 4.21 Human subjects

The investigation involving human subjects adhered to the principles outlined in the Declaration of Helsinki. Approval was obtained from Ethics Committees of Kumamoto University (approval No. 2671, No. 2640 and No. 2641) and Saga University (approval No. 2022-10-SCR-05, No. 2020-01-01, No. 2020-09-01), ensuring ethical oversight in all procedures involving human subjects. Informed written consent for participation was obtained through an opt-out procedure, allowing subjects the opportunity to decline inclusion in the study.

### 4.22 Echocardiographic analysis of human subjects

The study was approved by the Ethics Committees of Kumamoto University (approval No. 2671) and Saga University (approval No. 2022-10-SCR-05). A total of 28,260 patients enrolled at the Department of Cardiovascular Medicine, Saga University Hospital between January 2014 and November 2022 were underwent echocardiography (Supplementary Fig. 1). Two-dimensional echocardiography was performed using a ViVid 9 system (ViVid 9, GE Healthcare, Milwaukee, WI, USA) as described [46]. Measurements included LV end-diastolic dimension (LVD; d), LV end-systolic dimension (LVD; s) and left arterial dimension (LAD), wall thickness, early transmitral flow velocity (E), atrial systolic velocity (A) and early diastolic mitral annular velocity and early diastolic mitral annular velocity (e′). Percent fractional shortening (%FS) and left ventricular ejection fraction (LVEF) were calculated from left ventricular end-diastolic diameter (LVD; d) and end-systolic diameter (LVD; s), or from left ventricular end-diastolic/systolic volumes, which were calculated with LVD; d and LVD; s.

### 4.23 Immunohistochemical analysis

The study was approved by the Ethics Committees of Kumamoto University (approval No. No. 2640 and No. 2641) and Saga University (approval No. 2020-01-01, No. 2020-09-01). For this study, samples were fixed 24 h in 4% paraformaldehyde, paraffin-embedded, cut into 4-μm-thick sections, pretreated with periodic acid (Nichirei Biosciences), and then incubated overnight with anti-HINT1 antibody (1:400, ab124912, Abcam) at 4 °C. After PBS washing, specimens were incubated with goat anti-rabbit IgG conjugated with peroxidase (1:250, GE Healthcare Life Science) as a second antibody at room temperature for 60 min and counterstained with hematoxylin. Images were obtained using a BZ-X710 microscope (Keyence).

### 4.24 Statistics and reproducibility

Sample size and information relevant to statistical tests are reported in figure legends. No statistical methods were used to determine sample size, but sample sizes were determined based on previous reports. No exclusion/inclusion criteria were applied to mice used in this study. Group allocation and outcome assessment were performed in a blinded manner. *In vitro* experiments were repeated at least three times with similar results. All values were reported as the mean ± SD. Statistical analyses were performed using GraphPad Prism software (version 7.03, GraphPad Software). Data were assessed with two-group comparisons of variables using two-sided unpaired Student's *t*-test. Comparisons between three or more groups were performed using one-way ANOVA followed by Sidak's or Tukey's *post hoc* test. Mouse survival was analyzed by the Kaplan–Meier log-rank test. For all statistical analyses, a value of *p* < 0.05 was considered statistically significant.

## Fundings

This work was supported by the Scientific Research Fund of the Ministry of Education, Culture, Sports, Science and Technology (MEXT) of Japan (Grant 21H04825 to Y.O., Grant 23K07511 to M.S., Grant 25461114 to K.M., and Grant 23310135 to K.A.), the Core Research for Evolutional Science and Technology (CREST) Program of the Japan Agency for Medical Research and Development (AMED) (Grant 24gm1710006h0002 to Y.O.), the Takeda Science Foundation (2023, 2024) (M.S., T.K.), the SENSHIN Medical Research Foundation (2020) (M.S.), and a Grant for Basic Research of the Japanese Circulation Society (2020) (M.S.), the NAITO FOUNDATION (2021) (M.S.), the Mochida Memorial Foundation for Medical and Pharmaceutical Research (2021) (M.S.), the MSD Life Science Foundation (2021) (M.S.), a Japan Heart Foundation Research Grant (2022) (M.S.), the NOVARTIS Foundation (Japan) for the Promotion of Science (2022) (M.S.), the Ichiro Kanehara Foundation for the Promotion of Medical Sciences and Medical Care (2022) (M.S.), and the JGS (Japan Geriatrics Society) Research Grant Award in Geriatrics and Gerontology 2024 (M.S.). This research was partially supported by the Platform Project for Supporting Drug Discovery and Life Science Research (Basis for Supporting Innovative Drug Discovery and Life Science Research (BINDS)) from AMED under Grant Number JP23am121018 (support number 5755).

## CRediT authorship contribution statement

**Michio Sato:** Writing – review & editing, Writing – original draft, Visualization, Validation, Project administration, Methodology, Investigation, Funding acquisition, Formal analysis, Data curation, Conceptualization. **Tsuyoshi Kadomatsu:** Writing – review & editing, Writing – original draft, Visualization, Validation, Project administration, Methodology, Investigation, Funding acquisition, Formal analysis, Data curation, Conceptualization. **Jun Morinaga:** Validation, Formal analysis, Data curation. **Yuya Kinoshita:** Validation, Formal analysis, Data curation. **Daisuke Torigoe:** Validation, Formal analysis, Data curation. **Haruki Horiguchi:** Visualization, Validation, Data curation. **Sumio Ohtsuki:** Validation, Formal analysis, Data curation. **Shuji Yamamura:** Validation, Data curation. **Ryoko Kusaba:** Validation, Data curation. **Takanori Yamaguchi:** Resources. **Goro Yoshioka:** Validation, Resources, Data curation. **Kimi Araki:** Validation, Resources. **Tomohiko Wakayama:** Validation, Supervision, Formal analysis, Data curation. **Keishi Miyata:** Validation, Supervision, Data curation. **Koichi Node:** Validation, Supervision, Resources, Data curation. **Yuichi Oike:** Writing – review & editing, Writing – original draft, Validation, Supervision, Project administration, Methodology, Investigation, Funding acquisition, Conceptualization.

## Declaration of competing interest

The authors declare that they have no known competing financial interests or personal relationships that could have appeared to influence the work reported in this paper.

## Data Availability

Data will be made available on request.

## Appendix A Supplementary data

Multimedia component 3 Multimedia component 3

Multimedia component 6 **Supplementary F1. Echocardiography analysis of patients**. (A) Overview of echocardiography analysis of a total of 28,260 patients enrolled at the Department of Cardiovascular Medicine, Saga University hospital. Patients whose data represented outliers from the 1–99 percentiles in each echocardiographic parameter (echocardiographic index of one or more of IVST, LVPWT, LVEF, E/A, or SepE/e′ parameters) were excluded, and those numbers are shown in parentheses. (B) Overview of echocardiographic characteristics of patients. Values are shown as number and percentage of the categorical variable of gender and median and interquartile range (IQR) for continuous variables. IVST, interventricular septum thickness; LVPWT, left ventricular posterior wall thickness; LVD; s, left ventricular diameter at end systole; LVD; d, left ventricular diameter at end diastole; LVEF, left ventricular ejection fraction; E/A, early diastolic filling velocity/ atrial filling velocity ratio; sepE/e′, ratio of septum early diastolic filling velocity to early diastolic wall motion velocity. **Supplementary F2. Age-related changes in cardiac function seen in human subjects**. (A–F). Correlation of age with cardiac function in humans based on echocardiography. Left ventricular ejection fraction (LVEF) (A), ratio of transmitral flow velocity patterns by echocardiography for early transmitral flow velocity (E) to atrial systolic velocity (A) (E/A) (B), ratio E to early diastolic mitral annular velocity (e′) (E/e′) (C), interventricular septum thickness (IVST) (D), and left ventricular posterior wall thickness (LVPWT) (E). Spearman's rho and *P* values for correlations between each parameter and age are also shown (F). **Supplementary F3. Effects of calorie restriction on expression of genes associated with inflammation and mitochondrial biogenesis in aging mouse heart**. (A and B) Relative expression of genes associated with (A) inflammation and (B) mitochondrial biogenesis in hearts of 24-month-old mice fed ad libitum (AL) or subjected to 70% calorie restriction (CR) (*n* = 7, AL; *n* = 11, CR). AL values were set to 1. Statistical significance was determined by a two-sided unpaired Student's *t*-test. **Supplementary F4. Age-related changes in HINT1 protein levels seen in human subjects**. (A) Patient characteristics and background. (B) Immunohistochemical staining for HINT1 in heart tissues from atrium biopsy in cases of a young (34-year-old) non-heart failure (HF) patient (left), an aged (68-year-old) non-HF patient (middle) and a 60-year-old HF patient (right). Scale bars: 100 μm. Lower panels show magnified images of boxed regions in upper panels. Scale bars: 200 μm. **Supplementary F5. HINT1 does not interact with MITF or the β-catenin transcriptional complex**. (A) Immunoprecipitation (IP) using anti-Flag-tag antibody of HINT1 protein in HEK293 cells transfected singly or with both Flag-tagged HINT1 and HA-tagged MITF plasmids. Shown is representative immunoblotting of the Flag-tag (HINT1) and the HA-tag (MITF). Hsc70 served as loading control. (B) IP using anti-Flag-tag antibody of HINT1 protein in HEK293 cells transfected with Flag-tagged HINT1 or control plasmids. Shown is representative immunoblotting of Flag-tagged HINT1 and of Pontin and β-catenin. Hsc70 served as loading control. **Supplementary F6. Inhibition of the BAF complex decreases *Tfam* expression**. (A) IP of HINT1 protein in control and *Smarce1* KO P19.CL6 cell lines. Shown is representative immunoblotting for ARID1A, SMARCE1, BRG1, and HINT1. Hsc70 served as loading control. (B) Representative immunoblotting for ARID1A, BRG1, and SMARCE1 in C2C12 cells treated with or without 1 μM ACBI1. Hsc70 served as loading control. (C) Relative expression of *Tfam*, *Pgc1α*, *Nrf1*, and *Nrf2* in C2C12 cells treated with or without 1 μM ACBI1. (*n* = 5 per group) Values in the untreated (DMSO) group were set to 1. (D) Analysis of mtDNA content (left, *Cytb*/*B2m*; right, *Nd2*/*B2m*) in C2C12 cells treated with or without 1 μM ACBI1. (*n* = 6 per group) Values in the untreated (DMSO) group were set to 1. (E) Relative expression of *Pgc1α*, *Nrf1*, and *Nrf2* in hearts of indicated mice (*n* = 12, Young (3-month-old); n = 12, Aged (24-month-old)). Values in young mice were set at 1. Statistical significance was determined by a two-sided unpaired Student's *t*-test (C to E). n.s, not significant, between groups. **Supplementary F7. Generation of cardiac-specific *Hint1* KO mice**. (A) Schematic showing generation of *Hint1*^*Flox*/*Flox*^ mutant mice. (B) Representative gel image of PCR products used to determine the genotype of *Hint1*^+/+^, *Hint1*^*Flox*/+^, and *Hint1*^*Flox*/*Flox*^ mice. (C) Representative western blot (left) and quantification (right) of HINT1 protein levels in heart tissue of 25-month-old *Hint1*^*Flox*/*Flox*^; *αMHC*-*Cre* (CKO) and littermate *Hint1*^*Flox*/*Flox*^ (Control) mice (n = 8 per group). Hsc70 served as loading control. Values in control mice were set at 1. Statistical significance was determined by a two-sided unpaired Student's *t*-test. **Supplementary F8. Cardiac-specific *Hint1* KO mice show ameliorated age-related cardiac dysfunction and increased mitochondrial biogenesis in heart**. (A) Shown are representative M-mode echocardiography recordings in 25-month-old *Hint1*^*Flox*/*Flox*^ (Control) and *Hint1*^*Flox*/*Flox*^; *αMHC*-*Cre* (CKO) mice. (B) Left ventricular end-diastolic diameter (LVD; d) (mm) (left), left ventricular end-systolic diameter (LVD; s) (mm) (middle) and percent fractional shortening (%FS) (right) in indicated mice (*n* = 9, Control; *n* = 8, CKO). (C) Shown are representative transmitral flow velocity patterns determined from echocardiography for early transmitral flow velocity (E), atrial systolic velocity (A) (top row) and early diastolic mitral annular velocity (e′) (bottom row). (D) E/A (left) and E/e′ (right) ratios (*n* = 9, Control; *n* = 8, CKO). (E) Gross appearance of whole heart (top row; scale bar, 5 mm) and whole lung (bottom row; scale bar, 5 mm) in indicated groups. (F) Body weight (BW) (g) (left), and HW/BW (mg g^−1^) (middle) and lung weight per body weight (LW/BW) (mg g^−1^) (right) ratios in indicated mice (*n* = 8 per group). (G) Representative hematoxylin-eosin (HE)-staining in sections through the mid-portion of the heart (scale bar, 1 mm) in indicated mice. (H) Representative sections of left ventricle stained with wheat germ agglutinin (WGA) to indicate cardiomyocyte size in Control and CKO groups (left). Size distribution of myocardial cells (μm^2^) in indicated mice (right); number of cells = 279 (Control) and 281 (CKO). Scale bar, 100 μm. (I) Relative expression of genes associated with heart failure and fibrosis in heart tissues from indicated mice (*n* = 8 per group). Control group levels were set to 1. (J) Relative transcript levels of genes associated with mitochondrial biogenesis in heart tissue of indicated mice (*n* = 8 per group). Expression levels in the Control group were set to 1. (K) Relative mtDNA content (*Cytb*/*ActB*) in heart tissues of indicated groups (*n* = 8 per group). Values in the Control group were set to 1. Statistical significance was determined by a two-sided unpaired Student's *t*-test (B, D, F, and H to K). **Supplementary F9. Hearts of aged *Caren* Tg mice exhibit improved mitochondrial homeostasis and a decreased DNA damage response**. Shown are analyses of hearts from 24-month-old aged *Caren* Tg versus WT mice. (A) Representative western blots (left) and quantitation (right) of HINT1 in indicated groups (*n* = 7). Hsc70 served as loading control. Values in WT were set to 1. (B) Relative expression of genes associated with mitochondrial biogenesis in indicated groups (*n* = 8, WT; *n* = 7, CAG-*Caren* Tg). WT values were set to 1. (C) Representative western blots (left) and quantitation (right) of TFAM in indicated groups. Hsc70 served as loading control. Values in WT were set to 1. (D) Relative mtDNA content (*Cytb*/*ActB*) in heart tissues of indicated groups (*n* = 8, WT; *n* = 7, CAG-*Caren* Tg). (E) Representative immunohistochemical staining with 8-OHdG. Scale bar, 1 mm. (F) Representative western blot (left) and quantitation (right) of 4-HNE-modified protein levels in indicated heart tissues (*n* = 7 per group). Hsc70 served as loading control. Values in WT were set to 1. (G) Relative expression of genes associated with inflammation in indicated tissues (*n* = 8, WT; *n* = 7, CAG-*Caren* Tg). WT values were set to 1. Statistical significance was determined by a two-sided unpaired Student's *t*-test (A to D, F and G). **Supplementary F10. *Caren* overexpression in cardiomyocytes blocks age-related declines in cardiac function and improves exercise tolerance in aged mice**. (A) Shown are representative M-mode echocardiography recordings in 24-month-old WT and CAG-*Caren* transgenic (Tg) mice. (B) Left ventricular end-diastolic diameter (LVD; d) (mm) (left), left ventricular end-systolic diameter (LVD; s) (mm) (middle) and percent fractional shortening (%FS) (right) in indicated mice (*n* = 10, WT; *n* = 6, CAG-*Caren* Tg). (C) Shown are representative transmitral flow velocity patterns determined from echocardiography for early transmitral flow velocity (E), atrial systolic velocity (A) (top row) and early diastolic mitral annular velocity (e′) (bottom row). (D) E/A (left) and E/e′ (right) ratios (*n* = 8, WT; *n* = 6, CAG-*Caren* Tg). (E) Gross appearance of whole heart (top row; scale bar, 5 mm) and whole lung (bottom row; scale bar, 5 mm) in indicated groups. (F) Body weight (BW) (g) (left), and HW/BW (mg g^−1^) (middle) and lung weight per body weight (LW/BW) (mg g^−1^) (right) ratios in indicated mice (*n* = 8, WT; *n* = 6, CAG-*Caren* Tg). (G) Representative hematoxylin-eosin (HE)-staining in sections through the mid-portion of the heart (scale bar, 1 mm) in indicated genotypes. (H) Representative left ventricle sections from indicated genotypes stained with WGA to indicate cardiomyocyte size (left), and size distribution of myocardial cells (μm^2^) (*n* = 300 cells per group) (right). Scale bar, 100 μm. (I) Relative expression of genes associated with heart failure and fibrosis in heart tissues from indicated mice (*n* = 8, WT; *n* = 6, CAG-*Caren* Tg). Levels in WT were set to 1. (J) Treadmill analysis in indicated groups showing maximal speed (left), total exercise time (middle), and exercise distance (right) (*n* = 10, WT; *n* = 6, CAG-*Caren* Tg). Statistical significance was determined by a two-sided unpaired Student's *t*-test (B, D, F, and H to J). n.s, not significant, between groups. **Supplementary F11. Expression of genes associated with cardiac function, blood pressure, and heart rate in AAV6-*Caren*-treated aged mice**. (A) Systolic blood pressure (left) and heart rate (BPM) (right) in 24-month-old WT mice 2 months after injection with AAV6-*Caren* or AAV6-*GFP* (*n* = 22 per group). (B) Relative expression of genes associated with heart failure and fibrosis in heart tissue of 24-month-old WT mice 2 months after injection with AAV6-*Caren* or AAV6-*GFP* (*n* = 10–11 per group). Levels seen in the AAV6-*GFP* group were set to 1. Statistical significance was determined by a two-sided unpaired Student's *t*-test (A and B). n.s, not significant, between groups. **Supplementary F12. Aged mice show decreased mtDNA content in various tissues**. Relative mtDNA content (*Cytb*/*ActB*) in heart, kidney, liver, gastrocnemius and soleus muscle, and inguinal (iWAT) and epididymal (eWAT) white adipose tissues of 3- and 24-month-old WT mice (*n* = 10 per group). Levels seen in 3-month-old mice were set to 1. Statistical significance was determined by a two-sided unpaired Student's *t*-test. Multimedia component 6
