# Supplementary material for: HINT1 suppression protects against age-related cardiac dysfunction by enhancing mitochondrial biogenesis
Source: Mol Metab. 2025 Feb 3;93:102107. doi: 10.1016/j.molmet.2025.102107 (PMC11850129; doi:10.1016/j.molmet.2025.102107)
Supplement: Multimedia component 3 [file mmc3.docx]

**Supplementary Table 3. Primer pairs used for quantitative RT-PCR.**

| **Gene** |  | **Sequences** |
| --- | --- | --- |
| *Rps18* | Forward | TTCTGGCCAACGGTCTAGACAAC |
|  | Reverse | CCAGTGGTCTTGGTGTGCTGA |
| *Nppa* | Forward | GAGAGACGGCAGTGCTTCTAGGC |
|  | Reverse | CGTGACACACCACAAGGGCTTAGG |
| *Nppb* | Forward | AGGCGAGACAAGGGAGAACA |
|  | Reverse | GGAGATCCATGCCGCAGA |
| *Myh6* | Forward | CGCAATGCAGAGTCGGTGA |
|  | Reverse | TCCTGCAGCCGCATTAAGTTC |
| *Myh7* | Forward | CGGACCTTGGAAGACCAGAT |
|  | Reverse | GACAGCTCCCCATTCTCTGT |
| *Col1a1* | Forward | GAGCGGAGAGTACTGGATCGA |
|  | Reverse | CTGACCTGTCTCCATGTTGCA |
| *Ctgf* | Forward | CAAAGCAGCTGCAAATACCA |
|  | Reverse | GGCCAAATGTGTCTTCCAGT |
| *Nrf1* | Forward | TTGCCCAAGTGAATTACTCTGCTG |
|  | Reverse | TGCAGGACAGTCTGAGCCATC |
| *Nrf2* | Forward | TTGGCAGAGACATTCCCATTTGTA |
|  | Reverse | AGTCATGGCTGCCTCCAGAGA |
| *Pgc1α* | Forward | CCGTAAATCTGCGGGATGATG |
|  | Reverse | CAGTTTCGTTCGACCTGCGTAA |
| *Tfam* | Forward | TGAAGCTTGTAAATGAGGCTTGGA |
|  | Reverse | CGGATCGTTTCACACTTCGAC |
| *Prkn* | Forward | CGAAAGGAAACAAAATTGCCT |
|  | Reverse | ACGTCATTTCAGAAAGCCCA |
| *Tnfα* | Forward | AAGCCTGTAGCCCACGTCGTA |
|  | Reverse | GGCACCACTAGTTGGTTGTCTTTG |
| *Il-1β* | Forward | TCCAGGATGAGGACATGAGCAC |
|  | Reverse | GAACGTCACACACCAGCAGGTTA |
| *Il-6* | Forward | CCACTTCACAAGTCGGAGGCTTA |
|  | Reverse | GCAAGTGCATCATCGTTGTTCATAC |
| *Cxcl1* | Forward | CCGAAGTCATAGCCACACTCAA |
|  | Reverse | GCAGTCTGTCTTCTTTCTCCGTTAC |
| *Cxcl2* | Forward | CTCCTTTCCAGGTCAGTTAGC |
|  | Reverse | CAGAAGTCATAGCCACTCTCAA |
| *Cx3cl* | Forward | CCCTCGCCGCTCGCGTGGCTGCTGC |
|  | Reverse | AATGGCACGCTTGCCGCAGGACTCC |
| *Hint1* | Forward | AAATCCCCGCCAAGATCATC |
|  | Reverse | GGGGAAATGTCATGAAAAGC |
| *Caren* | Forward | GCATCATGACTGCCTGGG |
|  | Reverse | ATTTGTGTGTTCCTGGTGGG |

**Supplementary Table 4. Primer pairs used to quantify mitochondrial DNA content.**

| **Gene** |  | **Sequences** |
| --- | --- | --- |
| *CytB*  (Mitochondrial DNA) | Forward | GCTTTCCACTTCATCTTACCATTT |
|  | Reverse | TGTTGGGTTGTTTGATCCTG |
| *Nd2*  (Mitochondrial DNA) | Forward | TCCTCCTGGCCATCGTACTCAACT |
|  | Reverse | AGAAGTGGAATGGGGCGAGGC |
| *B2m*  (Nuclear DNA) | Forward | ATGGGAAGCCGAACATACTG |
|  | Reverse | CAGTCTCAGTGGGGGTGAA |
| *ActB*  (Nuclear DNA) | Forward | GGAAAAGAGCCTCAGGGCAT |
|  | Reverse | GAAGAGCTATGAGCTGCCTGA |

**Supplementary Table 5. Primer pairs used for CUT&RUN analysis.**

| **Gene** |  | **Sequences** |
| --- | --- | --- |
| *Tfam* | Forward | CCTGTCACCTTCTACCTTTCGG |
|  | Reverse | GTTCCGCGCCACAACTTCAG |
