# Supplementary material for: HINT1 suppression protects against age-related cardiac dysfunction by enhancing mitochondrial biogenesis
Source: Mol Metab. 2025 Feb 3;93:102107. doi: 10.1016/j.molmet.2025.102107 (PMC11850129; doi:10.1016/j.molmet.2025.102107)
Supplement: Multimedia component 6 — Supplementary F1. Echocardiography analysis of patients. (A) Overview of echocardiography analysis of a total of 28,260 patients enrolled at the Department of Cardiovascular Medicine, Saga University hospital. Patients whose data represented outliers from the 1–99 percentiles in each echocardiographic parameter (echocardiographic index of one or more of IVST, LVPWT, LVEF, E/A, or SepE/e′ parameters) were excluded, and those numbers are shown in parentheses. (B) Overview of echocardiographic characteristics of patients. Values are shown as number and percentage of the categorical variable of gender and median and interquartile range (IQR) for continuous variables. IVST, interventricular septum thickness; LVPWT, left ventricular posterior wall thickness; LVD; s, left ventricular diameter at end systole; LVD; d, left ventricular diameter at end diastole; LVEF, left ventricular ejection fraction; E/A, early diastolic filling velocity/ atrial filling velocity ratio; sepE/e′, ratio of septum early diastolic filling velocity to early diastolic wall motion velocity. Supplementary F2. Age-related changes in cardiac function seen in human subjects. (A–F). Correlation of age with cardiac function in humans based on echocardiography. Left ventricular ejection fraction (LVEF) (A), ratio of transmitral flow velocity patterns by echocardiography for early transmitral flow velocity (E) to atrial systolic velocity (A) (E/A) (B), ratio E to early diastolic mitral annular velocity (e′) (E/e′) (C), interventricular septum thickness (IVST) (D), and left ventricular posterior wall thickness (LVPWT) (E). Spearman's rho and P values for correlations between each parameter and age are also shown (F). Supplementary F3. Effects of calorie restriction on expression of genes associated with inflammation and mitochondrial biogenesis in aging mouse heart. (A and B) Relative expression of genes associated with (A) inflammation and (B) mitochondrial biogenesis in hearts of 24-month-old mice fed ad [file mmc6.pdf]

A

28,260 patients underwent echocardiography.

Patients whose data represented outliers from percentiles 1-99 in each echocardiographic parameter listed below were excluded.

1. IVST (n=290)
2. LVPWT (n=299)
3. LVEF (B-mode) (n=546)
4. E/A (n=452)
5. Sep E/e' (n=486)

The remaining 26,425 patients underwent complete case analysis.

B

Categorical variables			
		Number	Percent
Gender	Female	12,362	46.8
	Male	14,063	53.2

Continuous variables	N	Missing information	Median	IQR
Age	26,425		70	(60, 79)
IVST	26,276	149	10	(9, 11)
LVPWT	26,275	150	10	(9, 11)
LVDs	26,284	141	26	(23, 29)
LVDd	26,295	130	42	(38, 46)
LVEF (B mode)	25,734	691	69	(64, 74)
E/A	21,123	5,302	0.78	(0.64, 1.03)
Sep E/e'	20,999	5,426	10.3	(8.1, 13.2)

Total number of patients analyzed = 26,425

Supplementary Figure 1 Sato *et al.*

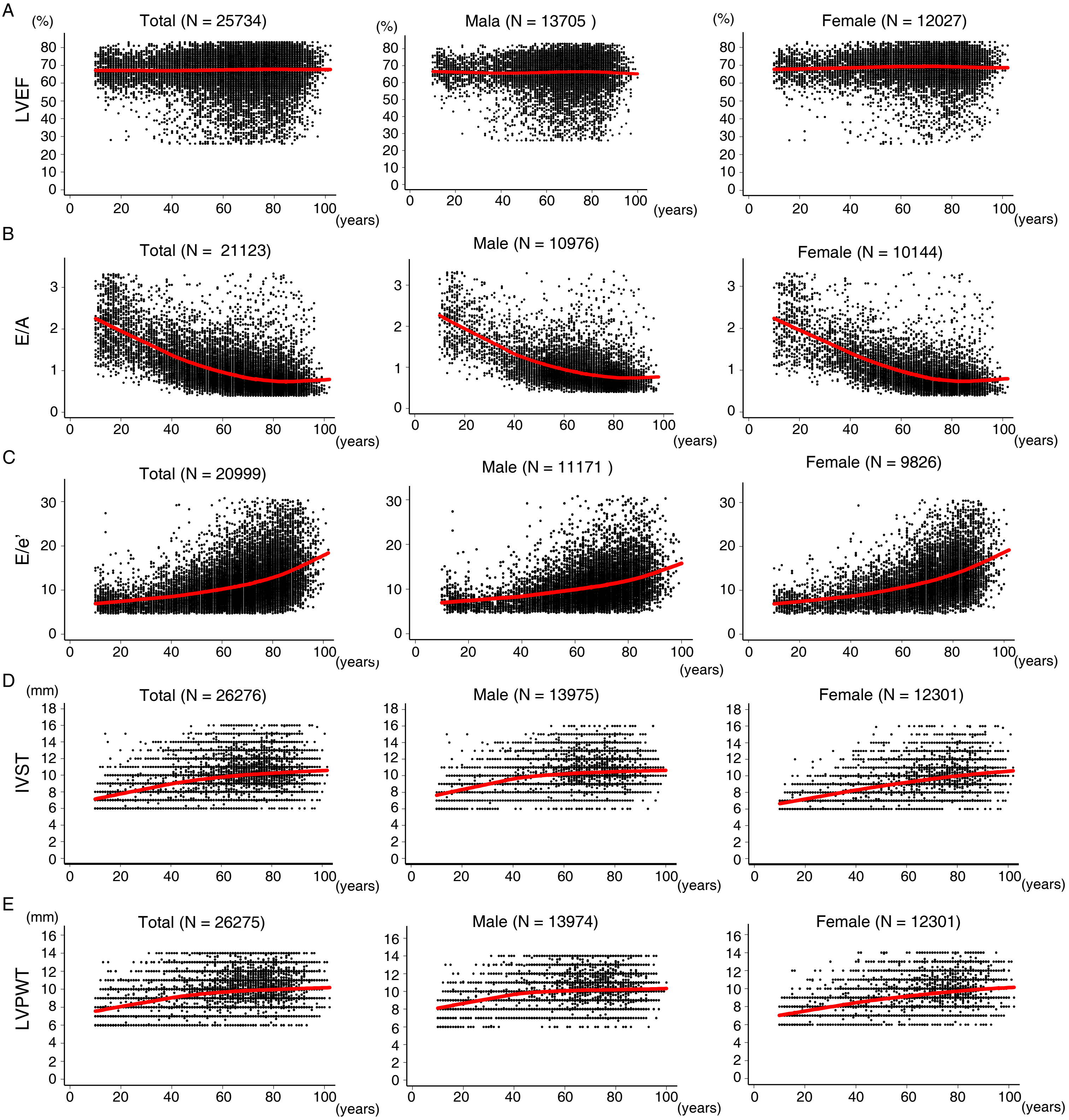

F

Parameter	Gender	Spearman's rho	P value
LVEF	Total	0.0391	<0.0001
	Male	0.0309	0.0003
	Female	0.0239	0.0088
E/A	Total	-0.527	<0.0001
	Male	-0.493	<0.0001
	Female	-0.5563	<0.0001
E/e'	Total	0.4473	<0.0001
	Male	0.3623	<0.0001
	Female	0.5215	<0.0001
IVST	Total	0.2677	<0.0001
	Male	0.187	<0.0001
	Female	0.4018	<0.0001
LVPWT	Total	0.2042	<0.0001
	Male	0.125	<0.0001
	Female	0.3385	<0.0001

Supplementary Figure 2 Sato *et al.*

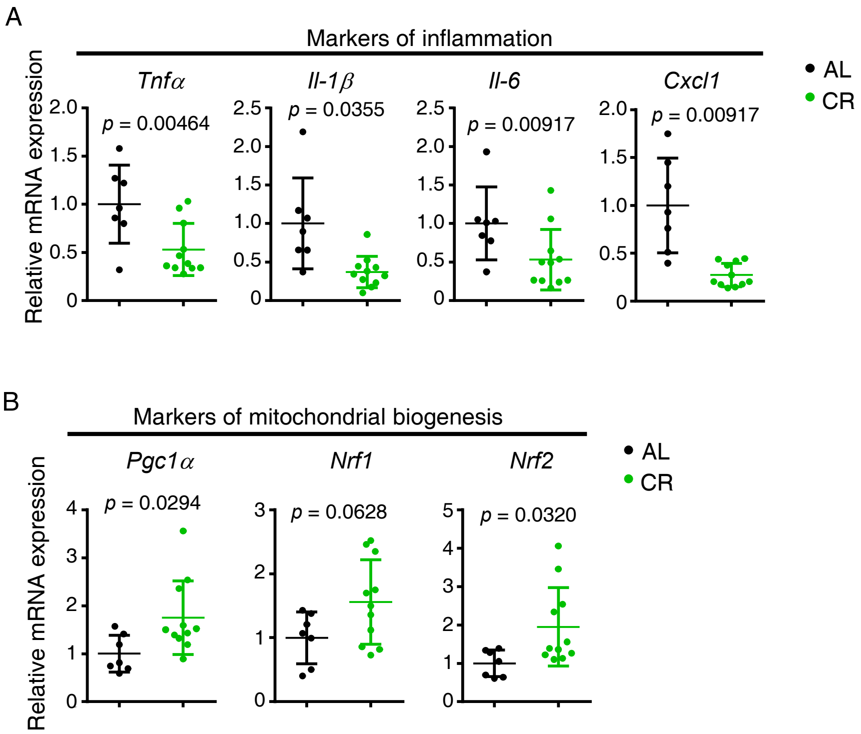
A
Markers of inflammation
Tnfα
Il-1β
Il-6
Cxcl1
AL
CR
p = 0.00464
p = 0.0355
p = 0.00917
p = 0.00917
Relative mRNA expression
B
Markers of mitochondrial biogenesis
Pgc1α
Nrf1
Nrf2
p = 0.0294
p = 0.0628
p = 0.0320
AL
CR
Relative mRNA expression

A

Case No	Sample ID	Age	Gender	Body mass index	NT-proBNP (pg/ml)	LVEF (modified simpson) (%)	E/e'	Underlying condition	Medication
Case1	H201141	34	M	25.1	26.4	63	6.25	paroxysmal atrial fibrillation, essential hypertension	Class Ⅰ antiarrhythmic drug
Case2	H201054	69	M	23.3	96.8	66	8.55	paroxysmal atrial fibrillation, essential hypertension, Past medical history of transient ischemic attack (TIA)	ARB, Ca channel blocker
Case3	H201022	60	M	23.9	1933	33	11.8	Chronic atrial fibrillation, Chronic heart failure, post surgical PVI and CTI	β-blocker

B

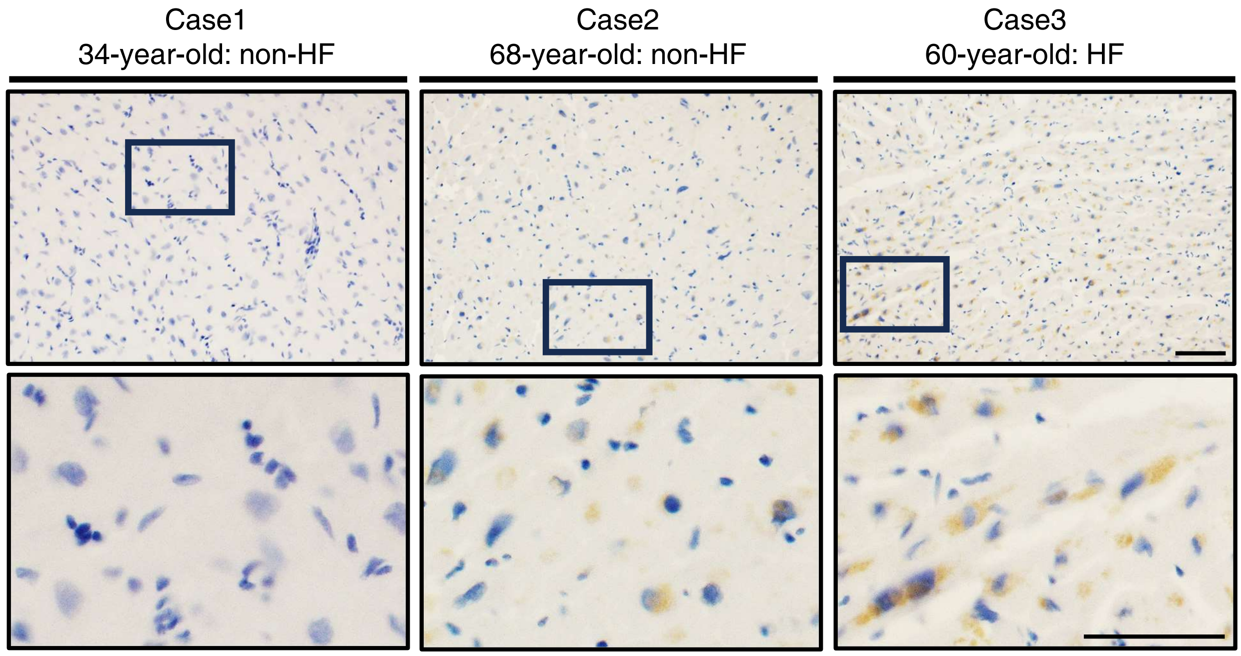

Supplementary Figure 4 Sato *et al*.

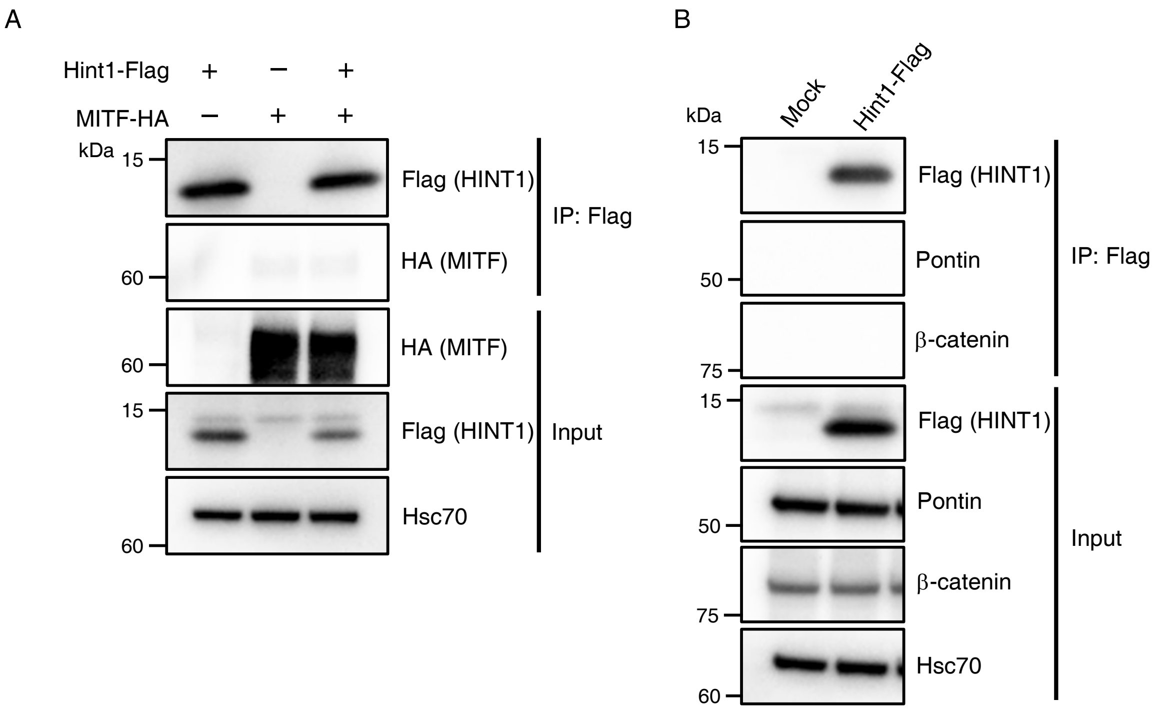

Supplementary Figure 5 Sato *et al*.

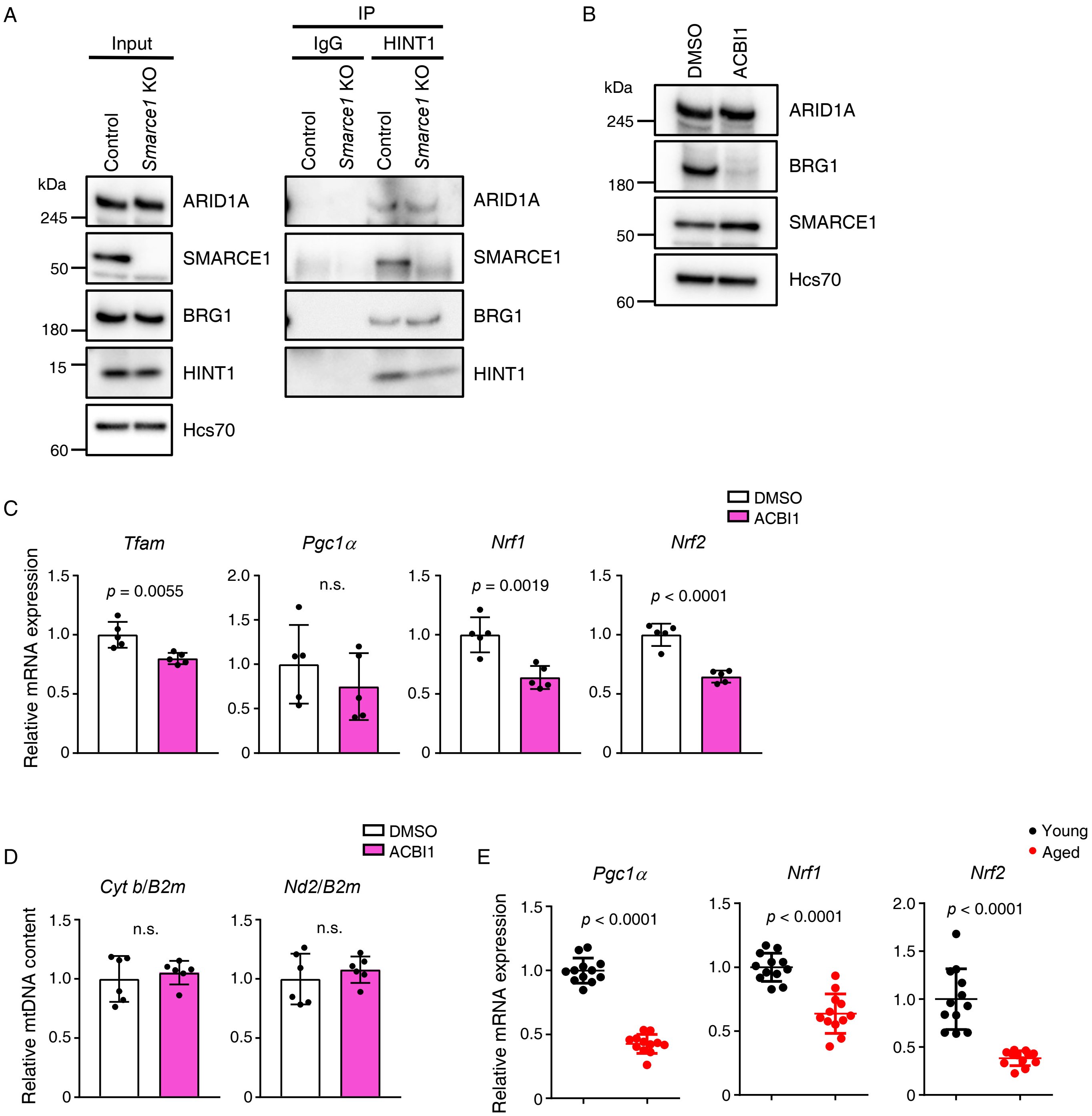
A
Input
IP
IgG
HINT1
Control
Smarce1 KO
kDa
245
50
180
15
60
ARID1A
SMARCE1
BRG1
HINT1
Hcs70
B
DMSO
ACBI1
kDa
245
180
50
60
ARID1A
BRG1
SMARCE1
Hcs70
C
DMSO
ACBI1
Tfam
Pgc1α
Nrf1
Nrf2
Relative mRNA expression
p = 0.0055
n.s.
p = 0.0019
p < 0.0001
D
DMSO
ACBI1
Cyt b/B2m
Nd2/B2m
Relative mtDNA content
n.s.
n.s.
E
Young
Aged
Pgc1α
Nrf1
Nrf2
Relative mRNA expression
p < 0.0001
p < 0.0001
p < 0.0001

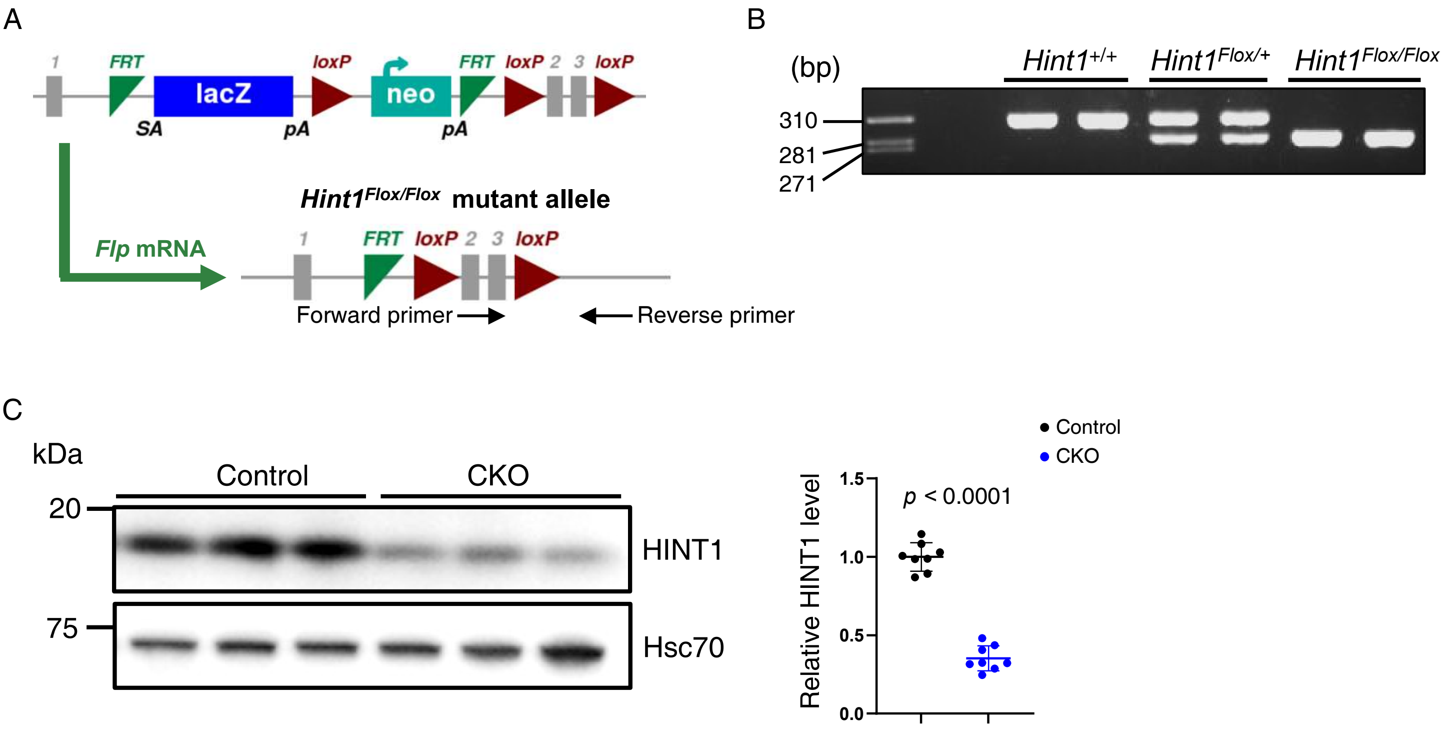
A
1
FRT
lacZ
loxP
neo
FRT
loxP
2
3
loxP
SA
pA
pA
Hint1Flox/Flox mutant allele
Flp mRNA
1
FRT
loxP
2
3
loxP
Forward primer
Reverse primer
B
(bp)
Hint1+/+
Hint1Flox/+
Hint1Flox/Flox
310
281
271
C
kDa
Control
CKO
20
HINT1
75
Hsc70
Control
CKO
p < 0.0001
Relative HINT1 level
1.5
1.0
0.5
0.0

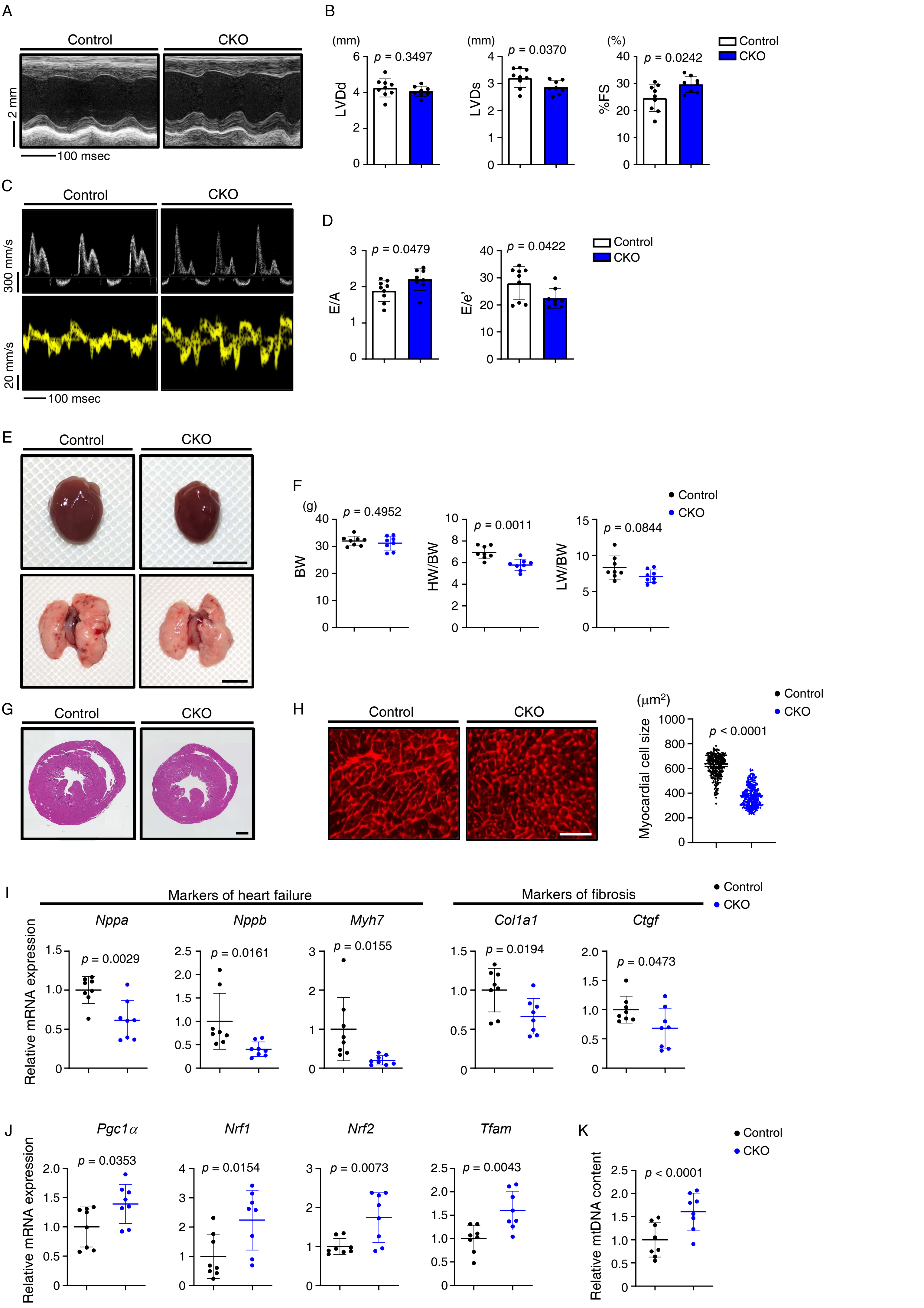

Supplementary Figure 8 Sato *et al.*

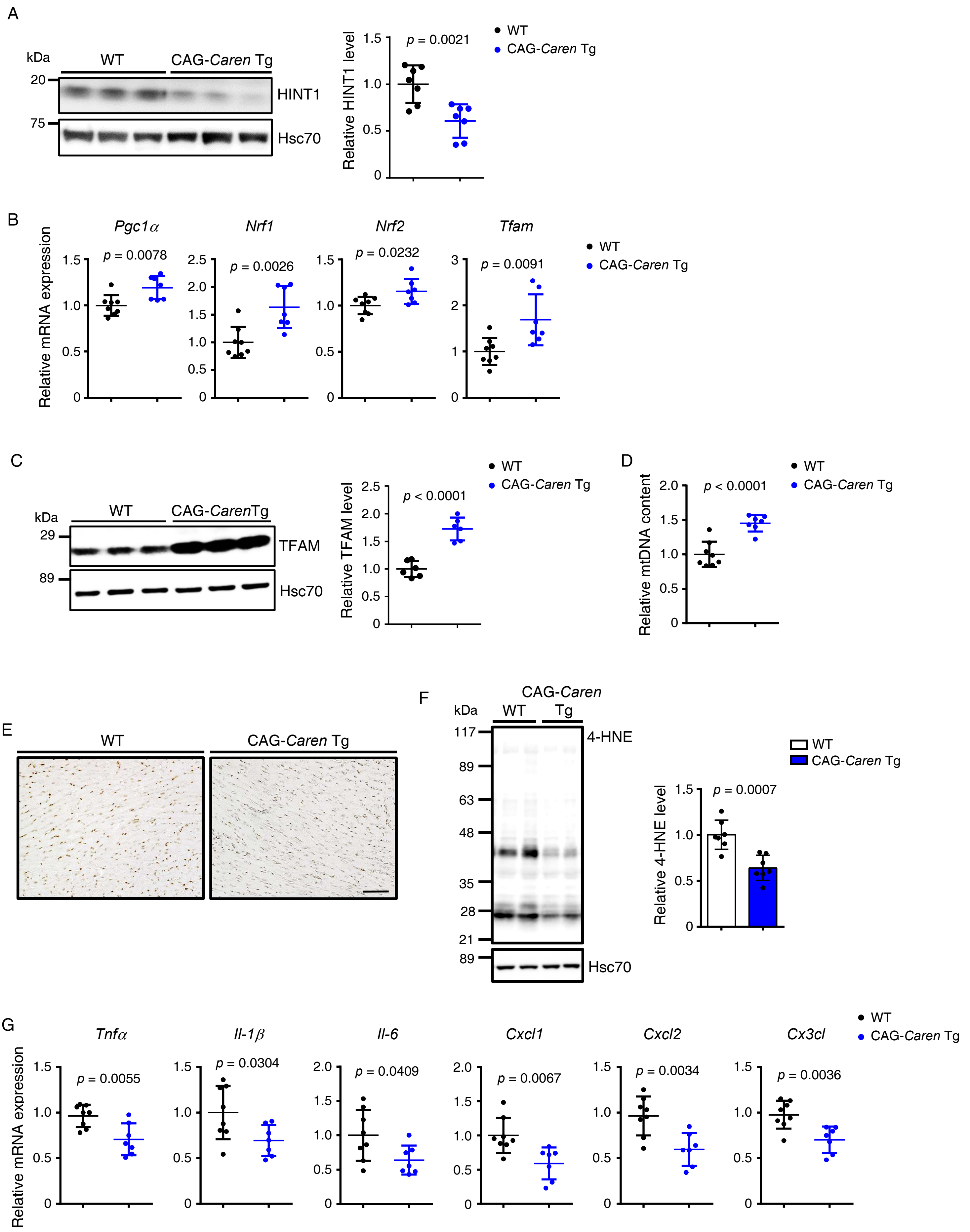

Supplementary Figure 9 Sato *et al.*

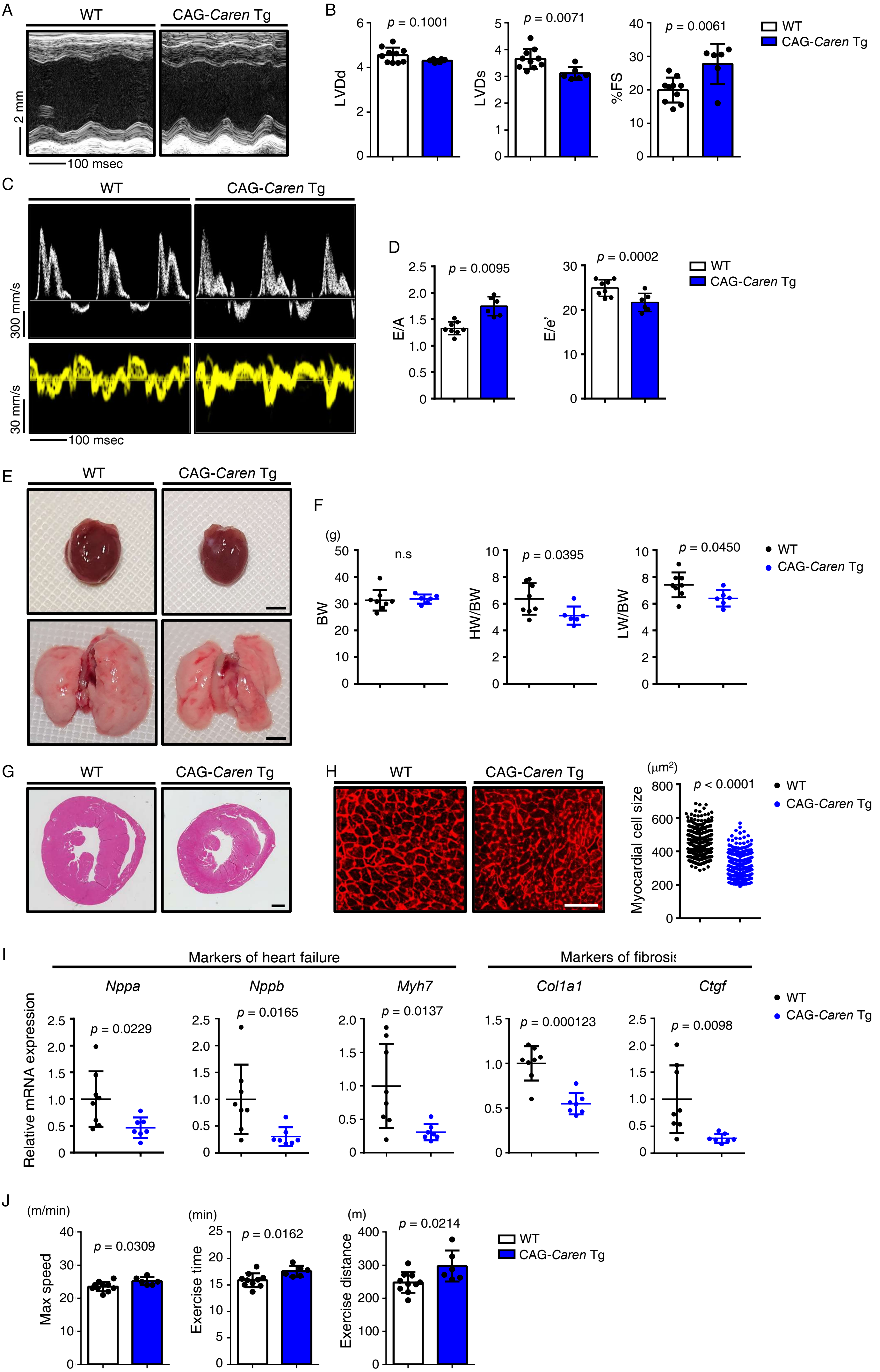

Supplementary Figure 10 Sato *et al*.

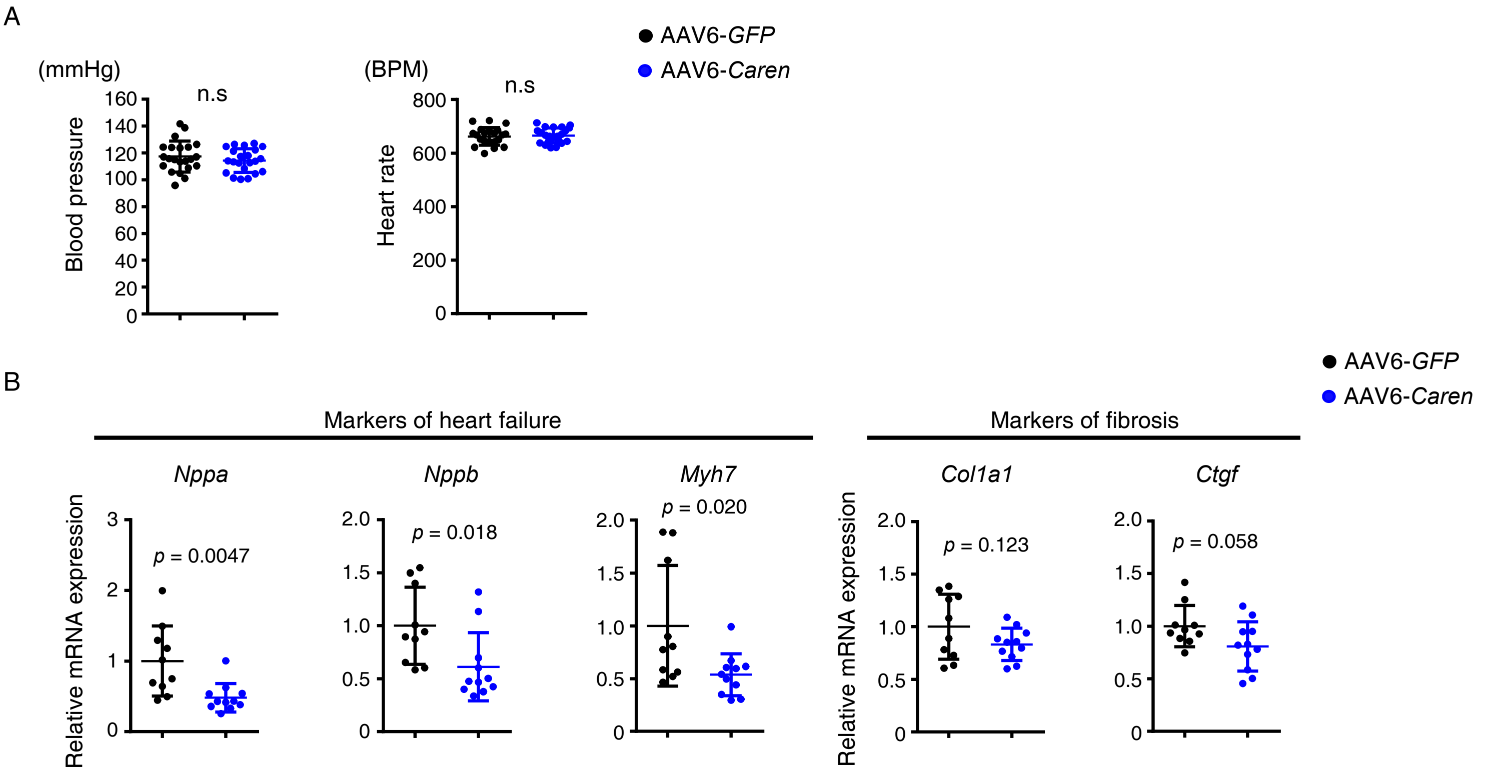
A
AAV6-GFP
AAV6-Caren
(mmHg)
n.s
Blood pressure
160
140
120
100
80
60
40
20
0
(BPM)
n.s
Heart rate
800
600
400
200
0
B
AAV6-GFP
AAV6-Caren
Markers of heart failure
Markers of fibrosis
Nppa
Nppb
Myh7
Col1a1
Ctgf
Relative mRNA expression
p = 0.0047
p = 0.018
p = 0.020
p = 0.123
p = 0.058

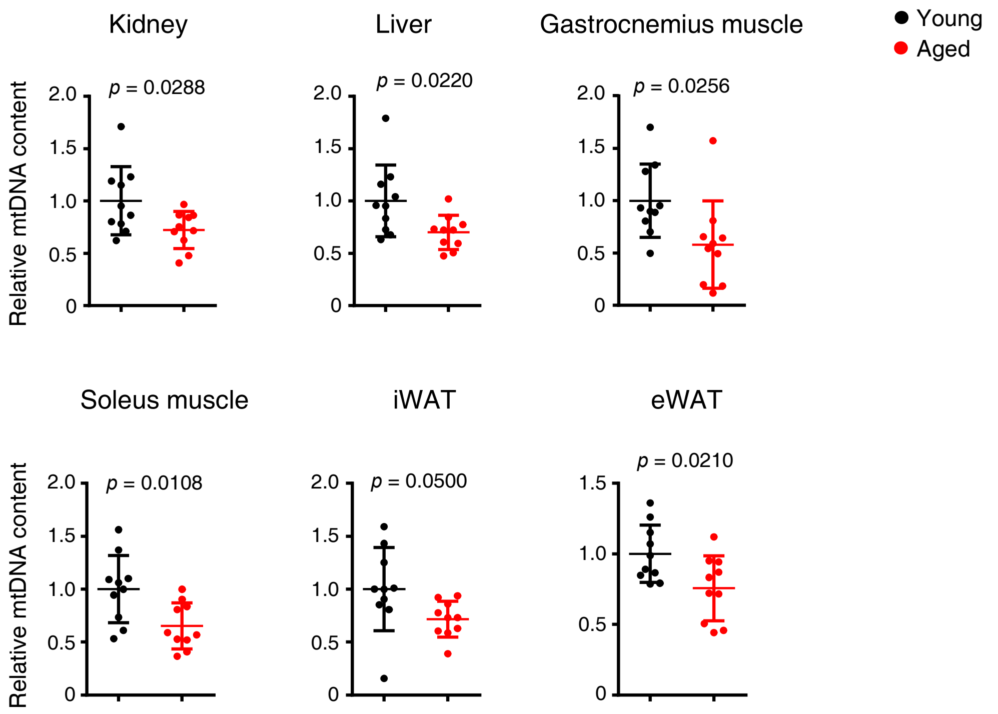

Supplementary Figure 12 Sato *et al*.
